# Pandoraviruses are highly derived phycodnaviruses

**DOI:** 10.1186/1745-6150-8-25

**Published:** 2013-10-23

**Authors:** Natalya Yutin, Eugene V Koonin

**Affiliations:** 1 National Center for Biotechnology Information, National Library of Medicine, National Institutes of Health, Bethesda, MD 20894, USA

## Abstract

The recently discovered Pandoraviruses are by far the largest viruses known, with their 2 megabase genomes exceeding in size the genomes of numerous bacteria and archaea. Pandoraviruses show a distant relationship with other nucleocytoplasmic large DNA viruses (NCLDV) of eukaryotes, lack some of the NCLDV core genes and in particular do not appear to be specifically related to the other, better characterized family of giant viruses, the Mimiviridae. Here we report phylogenetic analysis of 6 core NCLDV genes that confidently places Pandoraviruses within the family Phycodnaviridae, with an apparent specific affinity with Coccolithoviruses. We conclude that, despite their many unusual characteristics, Pandoraviruses are highly derived phycodnaviruses. These findings imply that giant viruses have independently evolved from smaller NCLDV on at least two occasions.

This article was reviewed by Patrick Forterre and Lakshminarayan Iyer. For the full reviews, see the Reviewers’ reports section.

## Findings

The discovery of giant viruses infecting unicellular eukaryotes, in particular amoeba, eliminated the distinction between viruses and cellular life forms in terms of size and genomic complexity [1]. Until very recently, all the discovered true giants of the virus world, with genomes exceeding 1 megabase (Mb) and encompassing more than 1,000 genes, were closely related members of the family Mimiviridae [2-4]. The gap between the members of the Mimiviridae and viruses outside this family was dramatic: apart from the mimiviruses, the largest viral genome, that of *Emiliana huxleyi* virus 86, was approximately 0.41 Mb in size [5]. The unexpected recent discovery of two strains of Pandoraviruses, *Pandoravirus salinus* and *Pandoravirus dulcis*, with genomes of at least 2.5 and 1.9 Mb, respectively, dramatically expanded the range of viral giantry [6]. In addition to being enormous, Pandoravirus genomes turned out to be highly unusual in that they showed little similarity to other viruses, lacked some of the core genes of the Nucleo-Cytoplasmic Large DNA Viruses (NCLDV, or the proposed order *Megavirales*) of eukaryotes [7-10] and failed to show clear-cut affinities in phylogenetic analysis [6]. We set out to investigate the repertoire of core NCLDV genes in pandoraviruses and their phylogenies in greater detail.

### Ancestral NCLDV genes in Pandoraviruses

**Figure 1:**
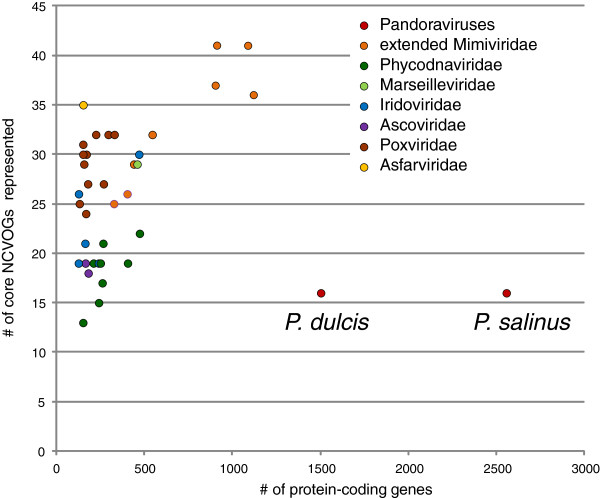
**Representation of Pandoraviruses and 7 NCLDV families in the NCVOGs vs the total number of (predicted) protein-coding genes.** ‘Extended Mimiviridae’ stands for Mimiviridae, *Cafeteria roenbergensis* virus, *Phaeocystis globosa* virus 12T, and Organic Lake phycodnaviruses that have been shown to comprise a monophyletic group [16].

The sequences of the predicted proteins of Pandoraviruses were compared to the sequences of the NCLDV included in the clusters of orthologous viral genes (NCVOGs) [11] resulting in the inclusion of Pandoraviruses in 67 NCVOGs (Additional file 1). In particular, we found that, of the 49 inferred ancestral genes (NCVOGs), only 17 were represented in one or both Pandoraviruses (Table 1). The low representation of Pandoraviruses in the NCVOGs and specifically, the absence of so many of the core, ancestral genes is anomalous among the NCLDV. To examine the extent of this anomaly, we tallied the number of ancestral NCVOGs that are represented in members of each of the 7 NCLDV families. The results indicate that Pandoraviruses stand out among the NCLDV with respect to the paucity of the (putative) ancestral viral genes (Figure 1). This lack of conservation of core NCLDV genes is all the more striking considering the huge genome size of Pandoraviruses compared to the other NCLDV (Figure 1) and suggests that Pandoraviruses are highly derived forms. Nevertheless, it should be stressed that the inclusion of Pandoraviruses into the NCLDV (in other words, their membership in the proposed order Megavirales [10]) is strongly supported by the presence of signature genes such as the primase-helicase fusion, packaging ATPase and thiol-disulfide oxidoreductase (Table 1). The obvious glaring gap in the repertoire of conserved genes in pandoraviruses is the absence of detectable capsid proteins. The most abundant virion proteins detected by proteomic analysis failed to show significant similarity to any known capsid proteins [6]. Furthermore, our attempts to identify putative derived capsid proteins by screening the pandoravirus protein sequences with position-specific scoring matrices obtained from multiple alignments of capsid proteins of different groups of NCLDV failed to identify any plausible candidates (data not shown).

**Table 1:** The ancestral NCLDV genes represented in Pandoraviruses

**Gene/NCVOG**	***P. dulcis *** **genes**	***P. salinus *** **genes**	**Presence in the 7 NCLDV families**^**a**^	**Best hit for Pandoraviruses**^**b **^**/ % identity/alignment length**
YqaJ viral recombinase family (pfam09588)/NCVOG1192	516302776	**516304314**	4	*Guillardia theta* CCMP2712 (Cryptophyta) /31/212
D5-like helicase-primase/NCVOG0023	516302795	**516304338**	7	*Bathycoccus* sp. RCC1105 virus BpV2 (Phycodnaviridae) /33/579
ribonucleoside diphosphate reductase, alpha subunit/NCVOG1353	516303570	**516306316**	5	*Thiothrix flexilis* (Proteobacteria) /36/1011
Ribonucleotide reductase small subunit; apparent eukaryotic origin/NCVOG0276	516302977	**516306307**	7	*Thalassiosira pseudonana* CCMP1335 (Stramenopiles) /75/216
dUTPase (cl00493)/NCVOG1068	516303522	**516305829**	3	*Hordeum vulgare* subsp. *vulgare* (Viridiplantae) /58/153
DNA or RNA helicases of superfamily II (COG1061) (A18hel)/NCVOG0076	516302732	**516304266**	5	*Ectocarpus siliculosus* virus 1 (Phycodnaviridae) /35/238
pfam04947, Poxvirus Late Transcription Factor VLTF3 like (A2L)/NCVOG0262	516302769, 516303263	**516304304,** 516305311	7	*Emiliania huxleyi* virus 202 (Phycodnaviridae) /34/264
Transcription factor S-II (TFIIS)-domain-containing protein/NCVOG0272	516303039	**516304685**	7	*Mus musculus* (Opisthokonta) /27/278
Nudix hydrolase (D10 ortholog)/NCVOG0236		**516305246**	5	*Vitis vinifera* (Viridiplantae) /28/226
A32-like packaging ATPase/NCVOG0249	516302762, 516303626	**516306303,** 516303793, 516305958, 516303807, 516305953	7	*Ostreococcus tauri* virus 2 (Phycodnaviridae) /45/247
uracil-DNA glycosylase/NCVOG1115	516303571	**516305889**	3	*Congregibacter litoralis* (Proteobacteria) /50/242
cd00127, DSPc, Dual specificity phosphatases (DSP); Ser/Thr and Tyr protein phosphatases/NCVOG0040	516303124, 516303141	**516304931,** 516304951	3	*Lausannevirus* (Marseillevirus family) /41/149
RING-finger-containing E3 ubiquitin ligase (COG5432: RAD18)/NCVOG0330	**516302773**		6	*Vitis vinifera* (Viridiplantae) /43/76
DNA-directed RNA polymerase subunit alpha/NCVOG0274	516302695	**516306301**	7	*Cyanidioschyzon merolae* strain 10D (Rhodophyta) /35/877
DNA-directed RNA polymerase subunit beta /NCVOG0271	516302872	**516306305**	7	*Capsella rubella* (Viridiplantae) /38/1283
disulfide (thiol) oxidoreductase; Erv1/Alr family (pfam04777)/NCVOG0052	516302900	**516304490**	7	*Setosphaeria turcica* Et28A (Opisthokonta) /37/123

^a)^The families are: Poxviridae, Asfarviridae, Iridoviridae, Ascoviridae, Marseilleviridae, Phycodnaviridae, Mimiviridae.

^b)^Genebank IDs of the genes for which the best hit is included are shown in bold.

### Phylogenetic analysis of conserved genes places Pandoraviruses within Phycodnaviridae

The pattern of best database hits in the BLASTP searches for the ancestral gene products of Pandoraviruses yielded a hint of a possible evolutionary relationship between Pandoraviruses and Phycodnaviridae, an expansive family of NCLDV that infect algae and other unicellular eukaryotes [12]. Indeed, among the best hits to homologous proteins from other NCLDV all but one were to homologs from the Phycodnaviridae family (Table 1).

**Figure 2:**
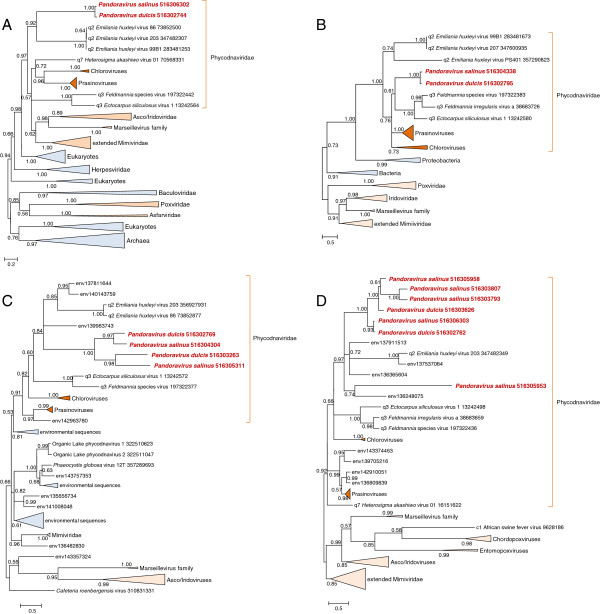
**Maximum-Likelihood trees of ancestral NCLDV genes present in Pandoraviruses. A**, DNA polymerase **B**, D5 primase-helicase. **C**, Poxvirus Late Transcription Factor VLTF3 like (A2L). **D**, A32-like packaging ATPase. Branches with bootstrap support less than 0.5 were collapsed. For individual sequences, the species name and the gene identification numbers are indicated; triangles denote multiple, collapsed sequences; env stands for environmental sequences (marine metagenome). Taxa abbreviations: c1, Asfarviridae; q2, Coccolithovirus; q3, Phaeovirus; q7, Raphidovirus.

**Figure 3:**
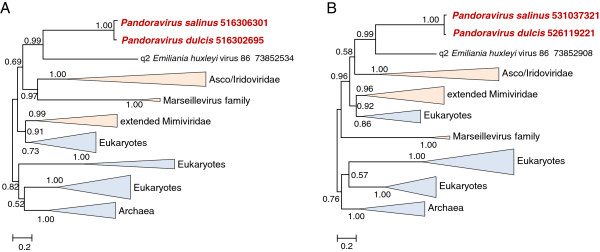
**Maximum-Likelihood trees of DNA-directed RNA polymerase. A**, alpha subunit. **B**, beta subunit. The designations are as in Figure 2.

To gain further insight into the origin of the Pandoraviruses, we then performed phylogenetic analysis of the 17 ancestral NCLDV genes that are represented in the pandoravirus genomes. In 6 of the 17 phylogenetic trees, Pandoraviruses grouped within the Phycodnaviridae clade, or in cases when such a clade was absent, with members of the family Phycodnaviridae (Figures 2-3 and Additional file 2). In 10 of the remaining trees, the Pandoravirus genes clustered with eukaryotic homologs (Additional file 2), suggestive of replacement of ancestral NCLDV genes with homologs derived from the hosts, as observed for multiple genes in the previous phylogenomic analysis of the NCLDV [13]. Only the gene for the dual specificity phosphatase (NCVOG0040) showed an apparent phylogenetic affinity with NCLDV outside Phycodnaviridae, namely with Marseilleviruses (Additional file 2). Similar to several other genes in the ancestral NCLDV gene set [13], the tree for the dual specificity phosphatases shows NCLDV scattered among homologs from cellular life forms (Additional file 2). This pattern suggests that the evolution of the phosphatase gene in the NCLDV involved multiple gene transfers and replacements. One of such gene transfers might have involved the phosphatase genes of pandoravirus and marseillevirus. Additional intervirus gene transfers could have involved among non-ancestral viral genes as implied by the detection of 17 pandoravirus genes with best database hits to mimivirus homologs [6]. Gene exchange between diverse viruses infecting amoebae has been reported previously. Indeed, amoebal cell, with their omnivorous phagocytic life style have been recognized as “melting pots” of horizontal gene transfers, so such intervirus gene exchanges could be expected.

Within the Phycodnaviridae, the preferred grouping of Pandoraviruses was with *Emiliana huxlei* virus (the type member of the genus Coccolithovirus [5]) as exemplified by the phylogenetic tree of the DNA polymerase, one of the most highly conserved genes of the NCLDV for which a reliable phylogeny can be obtained (Figure 2A). The highly conservative Approximately Unbiased (AU) test rejected all tested tree topologies with Pandoraviruses placed outside the *Phycodnaviridae* branch for the D5-like helicase-primase; for the other genes, some of the alternative topologies were not rejected by the AU test but all were assigned lower likelihood values (Additional file 2). Perhaps the strongest evidence of an evolutionary link between Pandoraviruses and Coccolithoviruses comes from the phylogenetic trees of two RNA polymerase (RNAP) subunits in which the two confidently grouped together as indicated by the bootstrap support value of 0.99 (Figure 3). Coccolithoviruses are the only genus of phycodnaviruses that encode the RNAP subunits; the rest of the phycodnaviruses have lost the ancestral RNAP genes, presumably because these viruses employ the host RNAP during a nuclear phase of their reproduction cycle [11,12]. Thus, the shared presence of the two monophyletic RNAP subunit genes in Pandoraviruses and Coccolithoviruses is a shared derived character that supports the common origin of these viruses.

Taken together, the phylogenetic analysis results indicate that the ancestral NCLDV genes in Pandoraviruses largely share the evolutionary history with the homologous genes of Phycodnaviruses, and more specifically, appear to have evolved from a common ancestor with Coccolithoviruses.

### Implications for the evolution of giant viruses

Despite their enormous size, Pandoraviruses show no evolutionary connection with the other family of giant viruses, the Mimiviridae. Instead, phylogenetic analysis of the ancestral NCLDV genes points to an affinity between Pandoraviruses and Phycodnaviruses. Moreover, Pandoraviruses appear to belong within the Phycodnavirus branch, being a sister group of Coccolithoviruses. Certainly, the phylogenomic analysis that leads to this conclusion involves a proverbial “tree of 1%” [14]. Indeed, the entire evidence hinges on the topologies of 6 phylogenetic trees, albeit those for key NLCDV genes, and on the finding that two RNAP subunits genes are shared between Pandoraviruses and Coccolithoviruses, to the exclusion of other Phycodnaviruses. However, given that altogether Pandoraviruses retain only 17 of the 49 inferred ancestral NCLDV genes, there is not much potential for obtaining additional evidence on the relationship between these viruses and the other NCLDV although, as noticed above, some interviral gene exchanges within amoeba might have occurred.

Thus, it appears that, despite their extremely unusual gene repertoires, Pandoraviruses are highly derived Phycodnaviruses. This conclusion implies that giant viruses have evolved independently from less complex NCLDV on at least two independent occasions, within the families Mimiviridae and Phycodnaviridae (Figure 2A). Given the much smaller genomes of the other NCLDV and the lack of substantial similarity between the gene repertoires of Pandoraviruses and Mimiviruses, the scenario of independent gain of numerous genes in two lineages of NCLDV appears much more plausible than the alternative that would involve extensive degradation of extremely complex ancestors in multiple lineages. The discovery of additional, perhaps independently evolving giant viruses appears likely, and identification of the aspects of virus biology that favor such dramatic genome expansions is of major interest.

## Conclusions

Phylogenomic analysis indicates that the giant Pandoraviruses, by far the largest viruses discovered to date, are highly derived Phycodnaviruses, most likely, the sister group of Coccolithoviruses. The more general implication of these findings is that giant viruses independently evolved in at least two lineages of the NCLDV.

## Methods

*P. dulcis* and *P. salinus* protein sequences were retrieved from the non-redundant database at the National Center for Biotechnology Information (NIH, Bethesda). The non-redundant protein sequence database was searched using the PSI-BLAST program [15], with default parameters and the predicted Pandoravirus protein sequences used as queries. The reported results reflect searchers performed in August, 2013. The sequences for phylogenetic analysis were collected using (i) BLAST searches against nr and environmental (env_nr) databases initiated by Pandoravirus protein sequences; (ii) the corresponding NCVOG sequences [11]; and (iii) the corresponding mimiCOG sequences [16]. Nearly identical sequences were eliminated using BLASTCLUST (http://www.ncbi.nlm.nih.gov/Web/Newsltr/Spring04/blastlab.html). Protein sequences were aligned using the MUSCLE program with default parameters [17]; columns containing a large fraction of gaps (greater than 30%) and non-homogenous columns defined as described previously [18] were removed from the alignment prior to phylogenetic analysis. A preliminary maximum-likelihood tree was constructed using the FastTree program with default parameters (JTT evolutionary model, discrete gamma model with 20 rate categories [19]) [19]. The preliminary tree and the alignment were then used to determine the best substitution matrix using Prottest [20]. Best matrices found by Prottest were as follows: LG+G (NCVOG0052, NCVOG1068, NCVOG0236, NCVOG0276, NCVOG0330, NCVOG1115), LG+G+F (NCVOG0249, NCVOG0040, NCVOG0023, NCVOG0076, NCVOG0038, NCVOG0274, NCVOG0271, NCVOG0262, NCVOG1353, NCVOG0272), and WAG+G+F (NCVOG1192). The final maximum-likelihood trees were constructed using TreeFinder (1,000 replicates, Search Depth 2), with the substitution matrix that was found to be the best for a given alignment [21]. The Expected-Likelihood Weights (ELW) of 1,000 local rearrangements were used as confidence values of TreeFinder tree branches [21]. For tree topology testing, whenever applicable, alternative (constrained) topologies were constructed and compared to the initial trees using TreeFinder [21]. Approximately unbiased (AU) test P value cutoff 0.05 was used for rejecting tree topologies [22].

## Reviewers’ reports

Reviewer 1: Patrick Forterre (Institut Pasteur)

Pandoraviruses are fascinating new organisms, which illustrates the capacity of viruses to produce drastically different types of virions, with strikingly different structures and genomes encoding from 2 genes up to 2500 genes [1]. In this paper, Yutin and Koonin have revisited the genomes of the two isolated Pandoraviruses and identified 6 of the 17 core NCLDV genes which consistently group within Phycodnaviridae (one of the NCLDV – or Megavirales – families) in phylogenetic analyses. They concluded that Pandoraviruses evolved from smaller Phycodnaviridae. The implication is that giant viruses (Mimiviridae and Pandoviruses) evolved twice independently from smaller viruses and not from cellular organisms.

The authors did not discuss the possibility that some Pandoravirus ancestor captured these 6 genes as an operon from a Phycodnavirus. We know that LGT can indeed occur between viruses co-infecting the same hosts. The authors state that: “in none of the trees pandoraviruses would cluster with any viruses outside the family Phycodnaviridae”. However, it seems that the dual specificity phosphatase NCVOG0040 branch with Mimiviridae (Lausannevirus and Marseillevirus) suggesting that LGT have indeed occurred between Pandoraviruses and Mimiviridae. In their paper, Philippe and co-workers mentioned the existence of 17 genes of P. salinus that have their closest homolog (34% identical residues in average) within the Megaviridae [6]. This seems in contradiction with the results reported here.

Authors’ response: *The exceptional case of the dual specificity phosphatase was overlooked in the original submission (although the tree was included in Additional file**2**), and we appreciate the reviewer pointing out this omission. Indeed, this case of apparent phylogenetic affinity between ancestral genes of Pandoraviruses and Marseilleviruses (sic! not Mimiviridae) is likely to originate from intervirus gene exchange within amoeba, and so do the non-ancestral genes apparently shared between Pandoraviruses and Mimiviruses. This aspect of the evolution of the giant viruses is briefly discussed in the revised manuscript. The full characterization of such gene exchanges requires a comprehensive phylogenomic analysis of giant viruses that is currently underway in our group. It should be noted, however, that ancestral genes of the NCLDV do not form operons or clusters, so the scenario under which pandoraviruses acquired the ancestral genes from Phycodnaviruses “as an operon” is hardly justified. More importantly, there is no contradiction between the conclusions of this work and the possibility of horizontal gene transfer between Pandoraviruses and Mimiviruses (and/or other viruses of amoeba) as the latter involved non-ancestral genes.*

The presence among the 6 core genes related to Phycodnavirus of the packaging ATPase typical of viruses whose major capsid protein (MCP) contains a double-jelly roll fold structure is intriguing, since such MCP has not been detected in Pandoraviruses. This suggests several possibilities:

1) Pandoraviruses do encode an MCP that share ancestry with that of Phycodnaviruses, but is highly divergent and cannot be detected by sequence similarity.

2) The structural proteins of Pandoraviruses are unrelated to those of NCLDV, but the detected ATPase is involved in packaging.

3) The structural proteins of Pandoraviruses involved in formation of the virion are unrelated to those of megavirales and the detected ATPase is not involved in packaging.

Could the authors discuss these different possibilities? Did they use sensitive methods to specifically search for MCP? Philippe et al. identified two abundant proteins that could be involved in formation of the virion. Did the authors analyse these proteins?

Authors’ response: *indeed, the absence of detectable capsid proteins in Pandoraviruses is most intriguing and is emphasized in the revised manuscript. Of the three hypotheses brought up in this comment, (1) and (2) appear to be most plausible. We did employ a sensitive search strategy to detect possible diverged capsid proteins homologous to those of other NCLDV as pointed out in the revised manuscript. With regard to the abundant virion proteins of pandoraviruses, we prefer to cite the original publication*[6]*. An exhaustive analysis of the sequences and predicted structures of these and other proteins of Pandoraviruses is a separate undertaking that will be published in due course.*

Viral lineages are better defined by their capsid proteins, because these proteins are hallmarks of viruses (I use here capsid in a broad definition, including all type of structural assemblage involved in the formation of a virion) [1]. It has been shown that viruses producing homologous capsids can use different types of replicons, and that exchanges of replicons cassette genes have rather frequently occurred between viruses [23]. At the moment, it is therefore a bit premature to definitely classify Pandoraviruses as an NCLDV, because we know nothing about their virion structural proteins. One could thus imagine that Pandoraviruses belong to a novel major viral lineage and recruited in the past a cassette of replication/transcription genes from a Phycodnavirus. However, this scenario, gene cassette shuffling. is especially prevalent in viruses with small DNA genomes and has never or rarely been observed in large DNA viruses. Could the authors comment on this last point?

Authors’ response: *The nature of viral “lineages” and the comparative utility of structural and replicative proteins for reconstructions of virus evolution are matters of a long, storied debate*[23-27]*. Probably, the key message is that viral evolution is a complex network of relationship that involves both numerous gene exchanges and intervals of vertical evolution of gene modules*[28,29]*. Accordingly, both structural proteins and replicative proteins are important for evolutionary reconstructions. As repeatedly argued, replicative proteins are more informative because they retain more sequence conservation, show a strong tendency to come in coevolving modules, and most crucially, provide the potential for reconstructing evolutionary relationships between viruses and capsid-less selfish elements. As demonstrated in detail elsewhere, such relationships are pervasive in the evolution of different classes of selfish agents and essential for understanding the routes of their evolution*[30]*. Under the weight of all these considerations, we stick to our classification of Pandoraviruses as bona fide members of the NCLDV (Megavirales). As for the transfer of cassettes of replicative genes, we are indeed unaware of such events in the evolution of NCLDV.*

My feeling is that the authors’s interpretation (independent evolution of “giant” viruses from “big” viruses) is the correct one, in agreement with previous suggestion that NCLDV originated from smaller viruses predating LUCA [31] and the recent accordion model for genome evolution of Megavirales proposed by Jonathan Filée [32]. However, it will be important to obtain more insights into the origin and history of other genes of Pandoraviruses, especially those involved in the formation of the virion.

Authors’ response: *we could not agree more*.

Anticipating criticisms, Yutin and Koonin remark that their analysis is a case of “tree of 1%” or less, since it is based on 7 genes only, out of 2500. However, one should not forget that the rRNA tree (0.1%) was sufficient to identify the three domains structure of the universal tree of life.

Authors’ response: *true but that criterion makes sense only because rRNA coevolves with numerous other genes, even if not perfectly*.

1) Philippe N, Legendre M, Doutre G, Couté Y, Poirot O, Lescot M, Arslan D, Seltzer V, Bertaux L, Bruley C, Garin J, Claverie JM, Abergel C. Pandoraviruses: amoeba viruses with genomes up to 2.5 Mb reaching that of parasitic eukaryotes. Science. 2013, 341:281-286.

2) Raoult D, Forterre P. Redefining viruses: lessons from Mimivirus. Nat Rev Microbiol. 2008, 6:315-319

3) Krupovic M, Bamford DH. Does the evolution of viral polymerases reflect the origin and evolution of viruses? Nat Rev Microbiol. 2009, 7:250;

4) Forterre P. Giant viruses: conflicts in revisiting the virus concept. Intervirology. 2010, 53:362-378.

5) Filée J. Route of NCLDV evolution: the genomic accordion. Curr Opin Virol. 2013 Jul 26. doi:pii: S1879-6257(13)00115-6. 10.1016/j.coviro.2013.07.00

Reviewer 2: Lakshminarayan Iyer (National Center for Biotechnology Information, National Library of Medicine, National Institutes of Health).

The giant Pandoraviruses are the largest dsDNA viruses sequenced to date with over 2000 genes. Although the initial sequencing effort recognized the relationship of the Pandoraviruses to the NCLDV, it did not clarify their precise affinities to other viruses within this group. Yutin and Koonin convincingly demonstrate that the Pandoraviruses are divergent Phycodnaviruses, and with the existing data posit a special relationship to Coccolithoviruses. The observations are independently reproducible and the conclusions justified given the data.

## Competing interests

The authors declare no conflict of interests.

## Author’s contributions

NY collected the data; NY and EVK analyzed the data; EVK wrote the manuscript which was read and approved by both authors.

## Supplementary Material

Additional file 1 The NCVOGs represented in Pandoraviruses. Click here for file

Additional file 2 Phylogenetic trees for the ancestral NCLDV genes present in Pandoraviruses and the AU test results. Click here for file

## References

[B1] Raoult D Forterre P Redefining viruses: lessons from Mimivirus Nat Rev Microbiol 2008 8 315 319 10.1038/nrmicro1858 18311164

[B2] Claverie JM Abergel C Ogata H Mimivirus Curr Top Microbiol Immunol 2009 8 89 121 10.1007/978-3-540-68618-7_3 19216436

[B3] Claverie JM Ogata H Audic S Abergel C Suhre K Fournier PE Mimivirus and the emerging concept of “giant” virus Virus Res 2006 8 1 133 144 10.1016/j.virusres.2006.01.008 16469402

[B4] Raoult D Audic S Robert C Abergel C Renesto P Ogata H La Scola B Suzan M Claverie JM The 1.2-megabase genome sequence of Mimivirus Science 2004 8 5700 1344 1350 10.1126/science.1101485 15486256

[B5] Wilson WH Schroeder DC Allen MJ Holden MT Parkhill J Barrell BG Churcher C Hamlin N Mungall K Norbertczak H Complete genome sequence and lytic phase transcription profile of a Coccolithovirus Science 2005 8 5737 1090 1092 10.1126/science.1113109 16099989

[B6] Philippe N Legendre M Doutre G Couté Y Poirot O Lescot M Arslan D Seltzer V Bertaux L Bruley C Pandoraviruses: amoeba Viruses with Genomes up to 2.5 Mb Reaching that of Parasitic Eukaryotes Science 2013 8 6143 281 286 10.1126/science.1239181 23869018

[B7] Iyer LM Aravind L Koonin EV Common origin of four diverse families of large eukaryotic DNA viruses J Virol 2001 8 23 11720 11734 10.1128/JVI.75.23.11720-11734.2001 11689653 PMC114758

[B8] Koonin EV Yutin N Origin and evolution of eukaryotic large nucleo-cytoplasmic DNA viruses Intervirology 2010 8 5 284 292 10.1159/000312913 20551680 PMC2895762

[B9] Colson P de Lamballerie X Fournous G Raoult D Reclassification of giant viruses composing a fourth domain of life in the new order Megavirales Intervirology 2012 8 5 321 332 10.1159/000336562 22508375

[B10] Colson P De Lamballerie X Yutin N Asgari S Bigot Y Bideshi DK Cheng XW Federici BA Van Etten JL Koonin EV “Megavirales”, a proposed new order for eukaryotic nucleocytoplasmic large DNA viruses Arch Virol 2013 2013 Jun 29. [Epub ahead of print] DOI: 10.1007/s00705-013-1768-6 10.1007/s00705-013-1768-6 PMC4066373 23812617

[B11] Yutin N Wolf YI Raoult D Koonin EV Eukaryotic large nucleo-cytoplasmic DNA viruses: clusters of orthologous genes and reconstruction of viral genome evolution Virol J 2009 8 223 10.1186/1743-422X-6-223 20017929 PMC2806869

[B12] Wilson WH Van Etten JL Allen MJ The Phycodnaviridae: the story of how tiny giants rule the world Curr Top Microbiol Immunol 2009 8 1 42 10.1007/978-3-540-68618-7_1 19216434 PMC2908299

[B13] Yutin N Koonin EV Hidden evolutionary complexity of Nucleo-Cytoplasmic Large DNA viruses of eukaryotes Virol J 2012 8 1 161 10.1186/1743-422X-9-161 22891861 PMC3493329

[B14] Dagan T Martin W The tree of one percent Genome Biol 2006 8 10 118 10.1186/gb-2006-7-10-118 17081279 PMC1794558

[B15] Altschul SF Madden TL Schaffer AA Zhang J Zhang Z Miller W Lipman DJ Gapped BLAST and PSI-BLAST: a new generation of protein database search programs Nucleic Acids Res 1997 8 17 3389 3402 10.1093/nar/25.17.3389 9254694 PMC146917

[B16] Yutin N Colson P Raoult D Koonin EV Mimiviridae: clusters of orthologous genes, reconstruction of gene repertoire evolution and proposed expansion of the giant virus family Virol J 2013 8 106 10.1186/1743-422X-10-106 23557328 PMC3620924

[B17] Edgar RC MUSCLE: multiple sequence alignment with high accuracy and high throughput Nucleic Acids Res 2004 8 5 1792 1797 10.1093/nar/gkh340 15034147 PMC390337

[B18] Yutin N Makarova KS Mekhedov SL Wolf YI Koonin EV The deep archaeal roots of eukaryotes Mol Biol Evol 2008 8 8 1619 1630 10.1093/molbev/msn108 18463089 PMC2464739

[B19] Price MN Dehal PS Arkin AP FastTree 2–approximately maximum-likelihood trees for large alignments PLoS One 2010 8 3 e9490 10.1371/journal.pone.0009490 20224823 PMC2835736

[B20] Darriba D Taboada GL Doallo R Posada D ProtTest 3: fast selection of best-fit models of protein evolution Bioinformatics 2011 8 8 1164 1165 10.1093/bioinformatics/btr088 21335321 PMC5215816

[B21] Jobb G von Haeseler A Strimmer K TREEFINDER: a powerful graphical analysis environment for molecular phylogenetics BMC Evol Biol 2004 8 18 10.1186/1471-2148-4-18 15222900 PMC459214

[B22] Shimodaira H An approximately unbiased test of phylogenetic tree selection Syst Biol 2002 8 3 492 508 10.1080/10635150290069913 12079646

[B23] Krupovic M Bamford DH Does the evolution of viral polymerases reflect the origin and evolution of viruses? Nat Rev Microbiol 2009 8 3 250 author reply 250 19198619 10.1038/nrmicro2030-c1

[B24] Koonin EV Senkevich TG Dolja VV The ancient virus world and evolution of cells Biol Direct 2006 8 1 29 10.1186/1745-6150-1-29 16984643 PMC1594570

[B25] Koonin EV Wolf YI Nagasaki K Dolja VV The complexity of the virus world Nat Rev Microbiol 2009 8 250 DOI:10.1038/nrmicro2030-c2

[B26] Krupovic M Bamford DH Virus evolution: how far does the double beta-barrel viral lineage extend? Nat Rev Microbiol 2008 8 941 948 10.1038/nrmicro2033 19008892

[B27] Krupovic M Bamford DH Double-stranded DNA viruses: 20 families and only five different architectural principles for virion assembly Curr Opin Virol 2011 8 2 118 124 10.1016/j.coviro.2011.06.001 22440622

[B28] Koonin EV Dolja VV A virocentric perspective on the evolution of life Curr Opin Virol 2013 8 5 546 557 10.1016/j.coviro.2013.06.008 23850169 PMC4326007

[B29] Krupovic M Networks of evolutionary interactions underlying the polyphyletic origin of ssDNA viruses Curr Opin Virol 2013 8 5 578 586 10.1016/j.coviro.2013.06.010 23850154

[B30] Koonin EV Dolja VV Virus world as an evolutionary network of viruses and capsid-less selfish elements Microbiol Mol Biol Rev 2014 in press 10.1128/MMBR.00049-13 PMC4054253 24847023

[B31] Forterre P Giant viruses: conflicts in revisiting the virus concept Intervirology 2010 8 5 362 378 10.1159/000312921 20551688

[B32] Filee J Route of NCLDV evolution: the genomic accordion Curr Opin Virol 2013 8 5 595 599 10.1016/j.coviro.2013.07.003 23896278

