# Supplementary material for: Pandoraviruses are highly derived phycodnaviruses
Source: Biol Direct. 2013 Oct 23;8:25. doi: 10.1186/1745-6150-8-25 (PMC3924356; doi:10.1186/1745-6150-8-25)
Supplement: Additional file 2 — Phylogenetic trees for the ancestral NCLDV genes present in Pandoraviruses and the AU test results. [file 1745-6150-8-25-S2.pptx]

## Slide 1
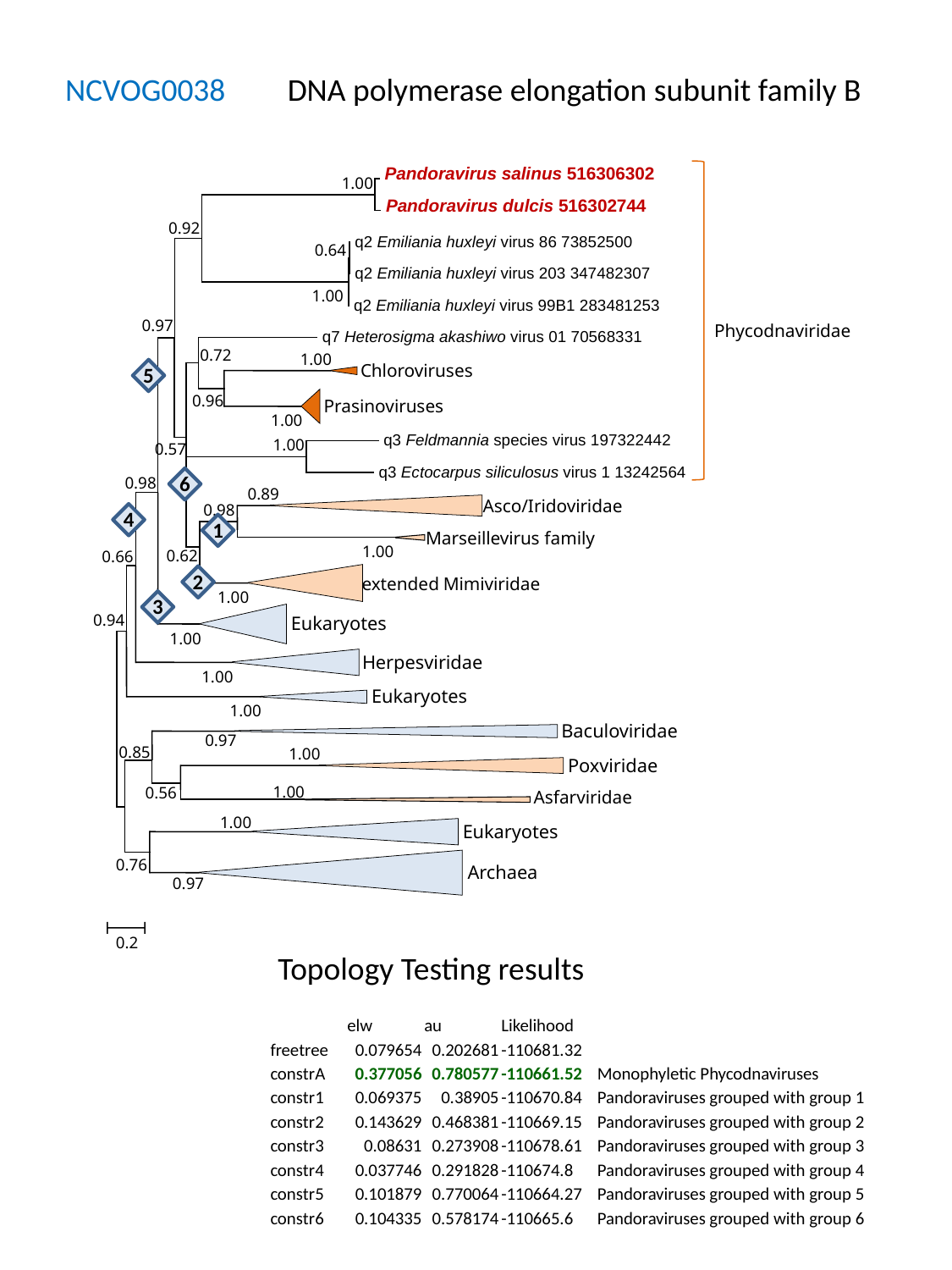

NCVOG0038
DNA polymerase elongation subunit family B
 Pandoravirus salinus 516306302
1.00
 Pandoravirus dulcis 516302744
0.92
 q2 Emiliania huxleyi virus 86 73852500
0.64
 q2 Emiliania huxleyi virus 203 347482307
1.00
 q2 Emiliania huxleyi virus 99B1 283481253
0.97
 Phycodnaviridae
 q7 Heterosigma akashiwo virus 01 70568331
0.72
1.00
 Chloroviruses
0.96
 Prasinoviruses
1.00
 q3 Feldmannia species virus 197322442
1.00
0.57
 q3 Ectocarpus siliculosus virus 1 13242564
0.98
0.89
 Asco/Iridoviridae
0.98
 Marseillevirus family
1.00
0.62
0.66
 extended Mimiviridae
1.00
0.94
 Eukaryotes
1.00
 Herpesviridae
1.00
 Eukaryotes
1.00
 Baculoviridae
0.97
0.85
1.00
 Poxviridae
1.00
0.56
 Asfarviridae
1.00
 Eukaryotes
0.76
 Archaea
0.97
0.2
5
6
4
1
2
3
Topology Testing results
| | elw | au | Likelihood | | | | |
| --- | --- | --- | --- | --- | --- | --- | --- |
| freetree | 0.079654 | 0.202681 | -110681.32 | | | | |
| constrA | 0.377056 | 0.780577 | -110661.52 | Monophyletic Phycodnaviruses | | | |
| constr1 | 0.069375 | 0.38905 | -110670.84 | Pandoraviruses grouped with group 1 | | | |
| constr2 | 0.143629 | 0.468381 | -110669.15 | Pandoraviruses grouped with group 2 | | | |
| constr3 | 0.08631 | 0.273908 | -110678.61 | Pandoraviruses grouped with group 3 | | | |
| constr4 | 0.037746 | 0.291828 | -110674.8 | Pandoraviruses grouped with group 4 | | | |
| constr5 | 0.101879 | 0.770064 | -110664.27 | Pandoraviruses grouped with group 5 | | | |
| constr6 | 0.104335 | 0.578174 | -110665.6 | Pandoraviruses grouped with group 6 | | | |

## Slide 2
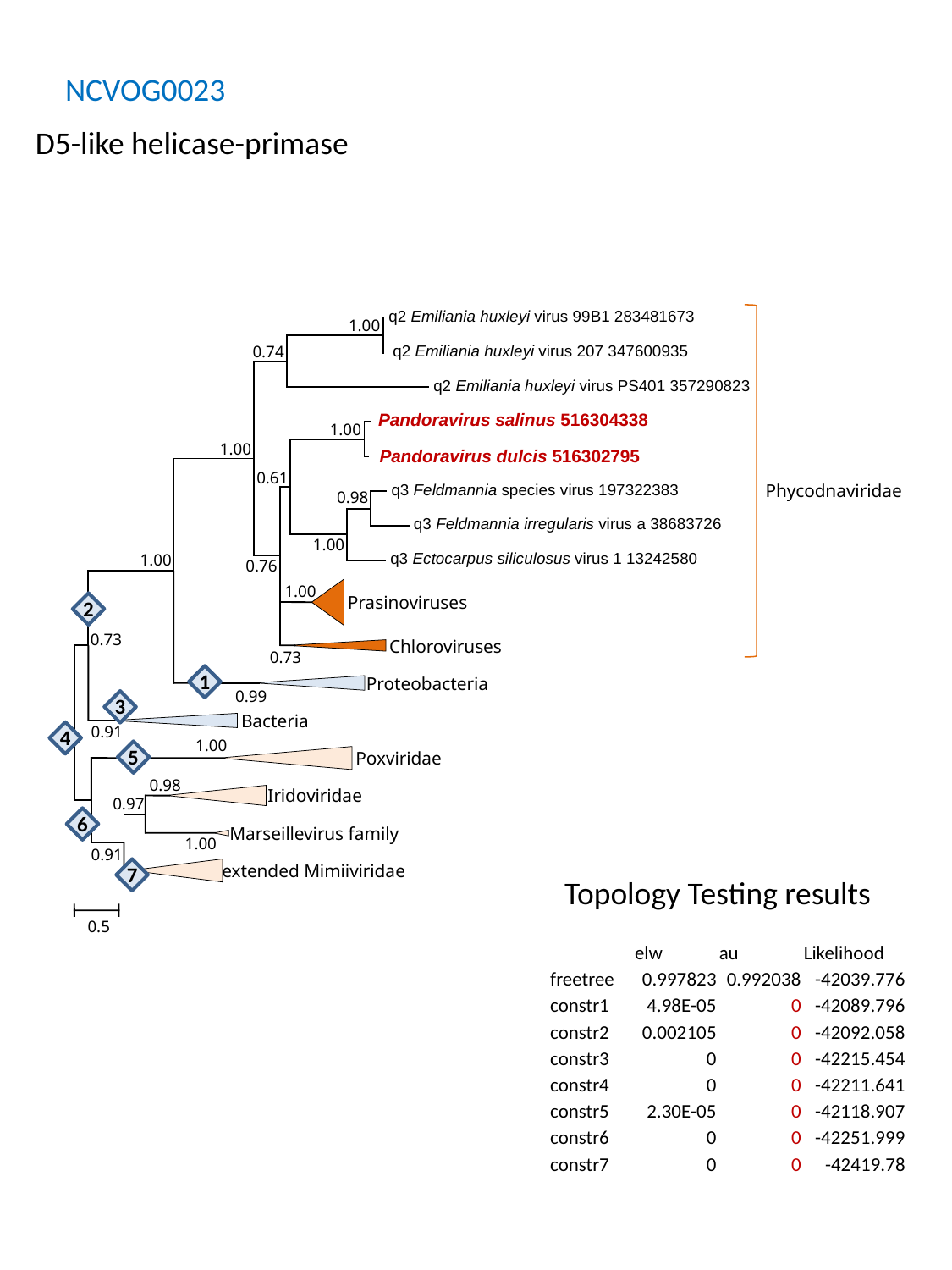

NCVOG0023
D5-like helicase-primase
 q2 Emiliania huxleyi virus 99B1 283481673
1.00
 q2 Emiliania huxleyi virus 207 347600935
0.74
 q2 Emiliania huxleyi virus PS401 357290823
 Pandoravirus salinus 516304338
1.00
1.00
 Pandoravirus dulcis 516302795
0.61
 q3 Feldmannia species virus 197322383
 Phycodnaviridae
0.98
 q3 Feldmannia irregularis virus a 38683726
1.00
 q3 Ectocarpus siliculosus virus 1 13242580
1.00
0.76
1.00
 Prasinoviruses
0.73
 Chloroviruses
0.73
 Proteobacteria
0.99
 Bacteria
0.91
1.00
 Poxviridae
0.98
 Iridoviridae
0.97
 Marseillevirus family
1.00
0.91
 extended Mimiiviridae
0.5
2
1
3
4
5
6
7
Topology Testing results
| | elw | au | Likelihood |
| --- | --- | --- | --- |
| freetree | 0.997823 | 0.992038 | -42039.776 |
| constr1 | 4.98E-05 | 0 | -42089.796 |
| constr2 | 0.002105 | 0 | -42092.058 |
| constr3 | 0 | 0 | -42215.454 |
| constr4 | 0 | 0 | -42211.641 |
| constr5 | 2.30E-05 | 0 | -42118.907 |
| constr6 | 0 | 0 | -42251.999 |
| constr7 | 0 | 0 | -42419.78 |

## Slide 3
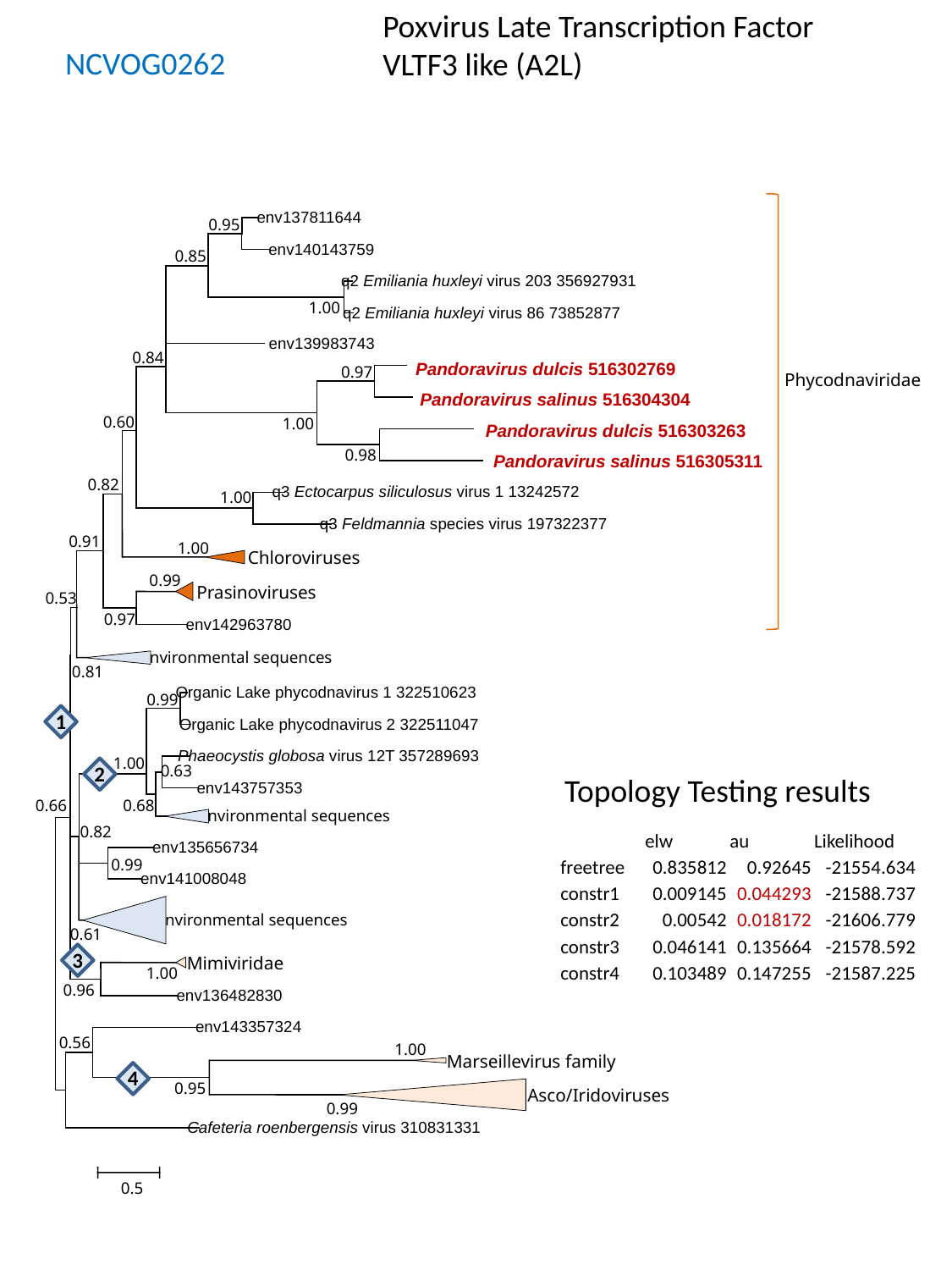

Poxvirus Late Transcription Factor VLTF3 like (A2L)
NCVOG0262
 env137811644
0.95
 env140143759
0.85
 q2 Emiliania huxleyi virus 203 356927931
1.00
 q2 Emiliania huxleyi virus 86 73852877
 env139983743
0.84
 Pandoravirus dulcis 516302769
0.97
 Phycodnaviridae
 Pandoravirus salinus 516304304
0.60
1.00
 Pandoravirus dulcis 516303263
0.98
 Pandoravirus salinus 516305311
0.82
 q3 Ectocarpus siliculosus virus 1 13242572
1.00
 q3 Feldmannia species virus 197322377
0.91
1.00
 Chloroviruses
0.99
 Prasinoviruses
0.53
0.97
 env142963780
 environmental sequences
0.81
 Organic Lake phycodnavirus 1 322510623
0.99
 Organic Lake phycodnavirus 2 322511047
 Phaeocystis globosa virus 12T 357289693
1.00
0.63
 env143757353
0.68
 environmental sequences
0.66
0.82
 env135656734
0.99
 env141008048
 environmental sequences
0.61
 Mimiviridae
1.00
0.96
 env136482830
 env143357324
0.56
1.00
 Marseillevirus family
0.95
 Asco/Iridoviruses
0.99
 Cafeteria roenbergensis virus 310831331
0.5
1
2
3
4
Topology Testing results
| | elw | au | Likelihood |
| --- | --- | --- | --- |
| freetree | 0.835812 | 0.92645 | -21554.634 |
| constr1 | 0.009145 | 0.044293 | -21588.737 |
| constr2 | 0.00542 | 0.018172 | -21606.779 |
| constr3 | 0.046141 | 0.135664 | -21578.592 |
| constr4 | 0.103489 | 0.147255 | -21587.225 |

## Slide 4
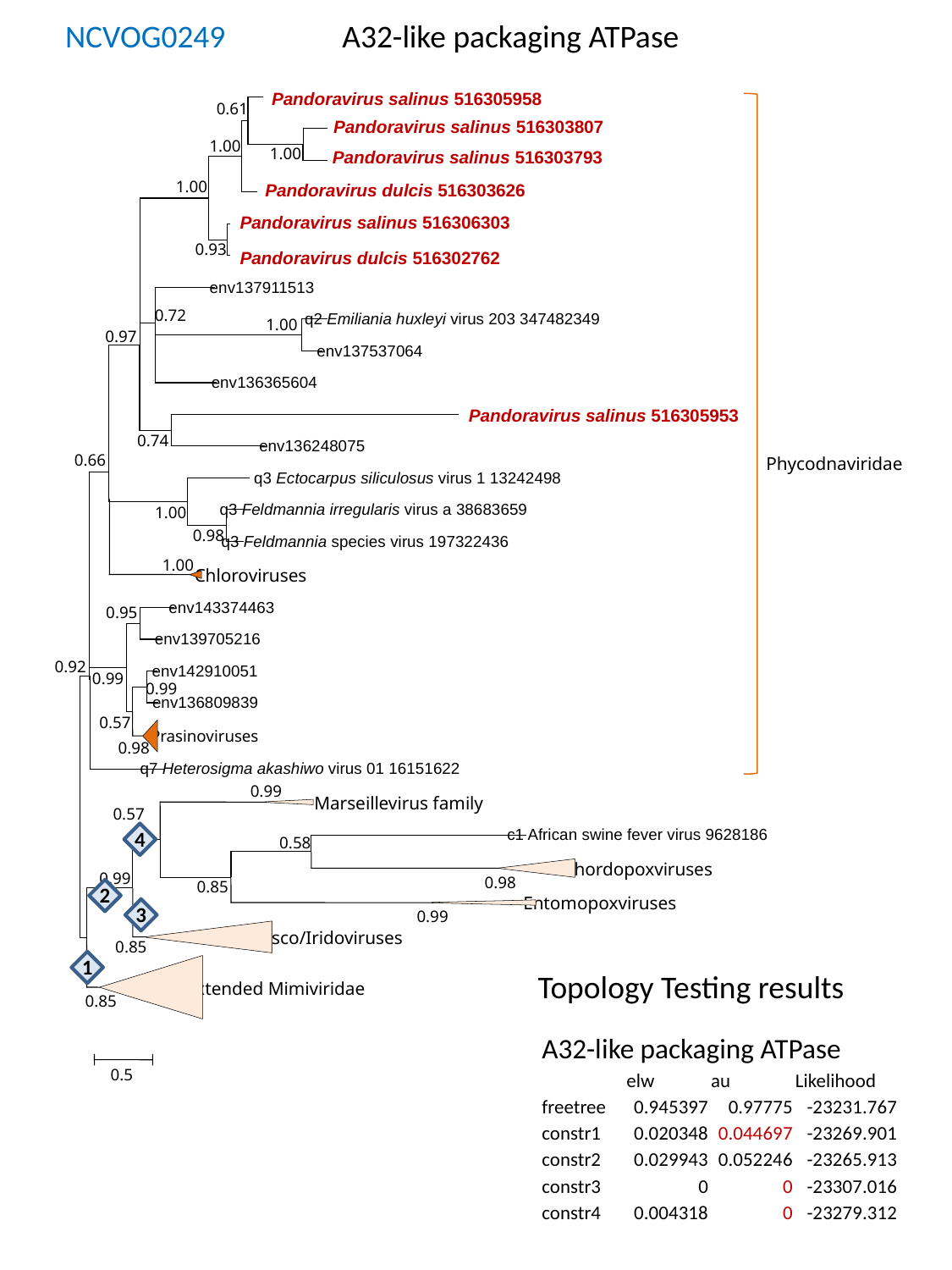

NCVOG0249
A32-like packaging ATPase
 Pandoravirus salinus 516305958
0.61
 Pandoravirus salinus 516303807
1.00
1.00
 Pandoravirus salinus 516303793
1.00
 Pandoravirus dulcis 516303626
 Pandoravirus salinus 516306303
0.93
 Pandoravirus dulcis 516302762
 env137911513
0.72
 q2 Emiliania huxleyi virus 203 347482349
1.00
0.97
 env137537064
 env136365604
 Pandoravirus salinus 516305953
0.74
 env136248075
0.66
 Phycodnaviridae
 q3 Ectocarpus siliculosus virus 1 13242498
 q3 Feldmannia irregularis virus a 38683659
1.00
0.98
 q3 Feldmannia species virus 197322436
1.00
 Chloroviruses
 env143374463
0.95
 env139705216
0.92
 env142910051
0.99
0.99
 env136809839
0.57
 Prasinoviruses
0.98
 q7 Heterosigma akashiwo virus 01 16151622
0.99
 Marseillevirus family
0.57
 c1 African swine fever virus 9628186
0.58
 Chordopoxviruses
0.99
0.98
0.85
 Entomopoxviruses
0.99
 Asco/Iridoviruses
0.85
 extended Mimiviridae
0.85
0.5
4
2
3
1
Topology Testing results
| A32-like packaging ATPase | | | |
| --- | --- | --- | --- |
| | elw | au | Likelihood |
| freetree | 0.945397 | 0.97775 | -23231.767 |
| constr1 | 0.020348 | 0.044697 | -23269.901 |
| constr2 | 0.029943 | 0.052246 | -23265.913 |
| constr3 | 0 | 0 | -23307.016 |
| constr4 | 0.004318 | 0 | -23279.312 |

## Slide 5
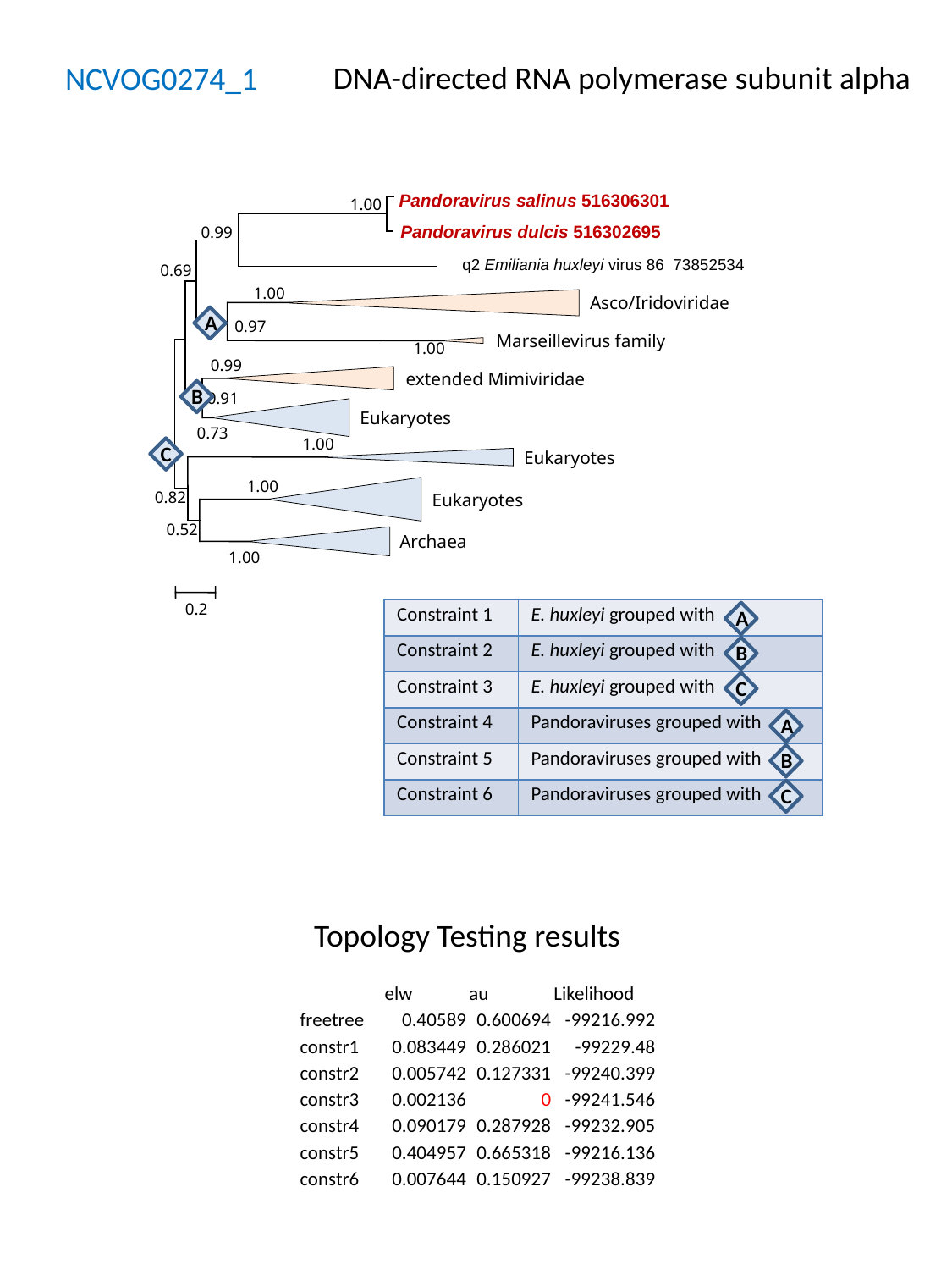

DNA-directed RNA polymerase subunit alpha
NCVOG0274_1
 Pandoravirus salinus 516306301
1.00
 Pandoravirus dulcis 516302695
0.99
 q2 Emiliania huxleyi virus 86 73852534
0.69
1.00
 Asco/Iridoviridae
0.97
 Marseillevirus family
1.00
0.99
 extended Mimiviridae
0.91
 Eukaryotes
0.73
1.00
 Eukaryotes
1.00
0.82
 Eukaryotes
0.52
 Archaea
1.00
0.2
A
B
C
A
B
C
| Constraint 1 | E. huxleyi grouped with |
| --- | --- |
| Constraint 2 | E. huxleyi grouped with |
| Constraint 3 | E. huxleyi grouped with |
| Constraint 4 | Pandoraviruses grouped with |
| Constraint 5 | Pandoraviruses grouped with |
| Constraint 6 | Pandoraviruses grouped with |
A
B
C
Topology Testing results
| | elw | au | Likelihood |
| --- | --- | --- | --- |
| freetree | 0.40589 | 0.600694 | -99216.992 |
| constr1 | 0.083449 | 0.286021 | -99229.48 |
| constr2 | 0.005742 | 0.127331 | -99240.399 |
| constr3 | 0.002136 | 0 | -99241.546 |
| constr4 | 0.090179 | 0.287928 | -99232.905 |
| constr5 | 0.404957 | 0.665318 | -99216.136 |
| constr6 | 0.007644 | 0.150927 | -99238.839 |

## Slide 6
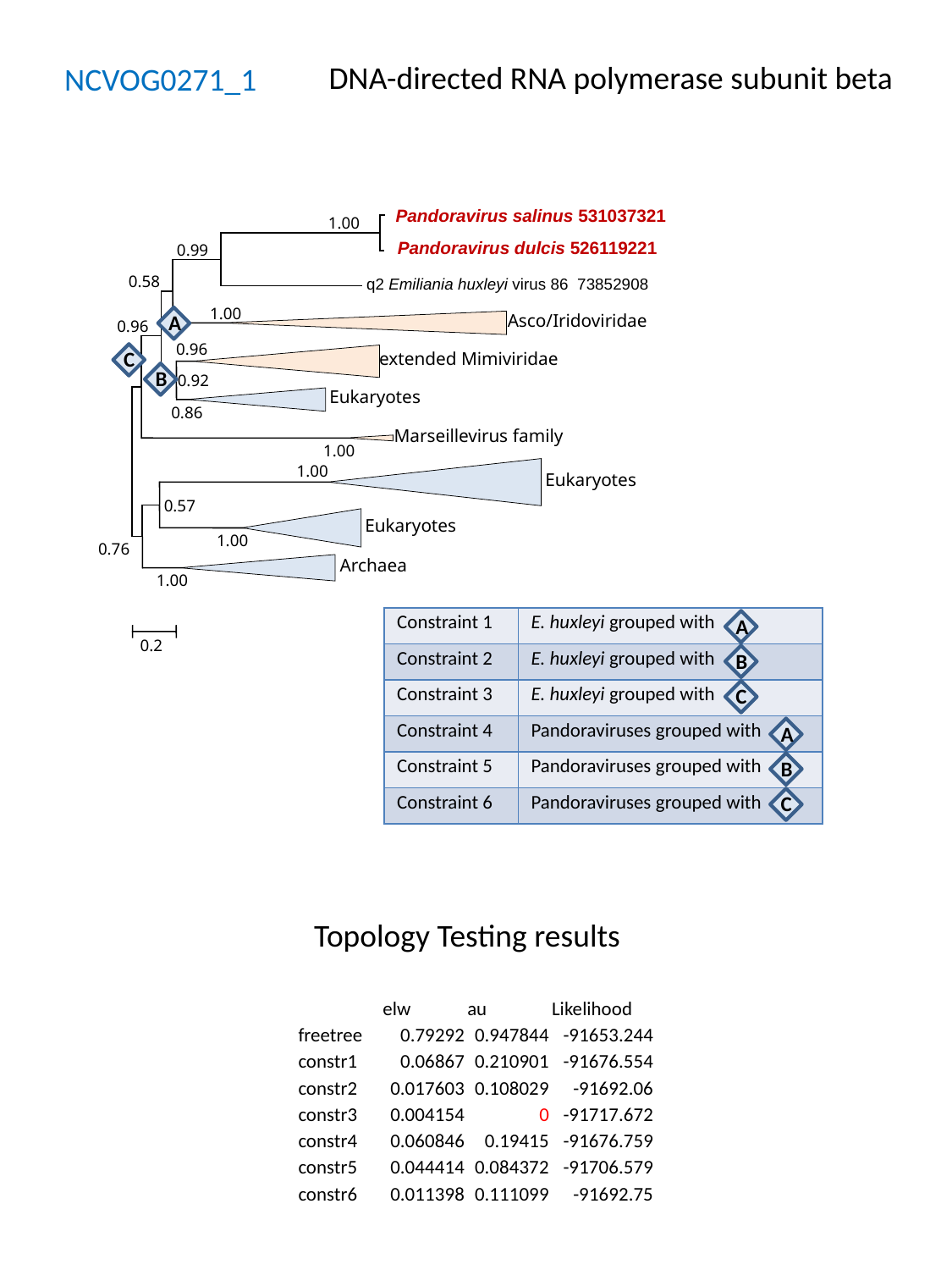

DNA-directed RNA polymerase subunit beta
NCVOG0271_1
 Pandoravirus salinus 531037321
1.00
 Pandoravirus dulcis 526119221
0.99
0.58
 q2 Emiliania huxleyi virus 86 73852908
1.00
 Asco/Iridoviridae
0.96
0.96
 extended Mimiviridae
0.92
 Eukaryotes
0.86
 Marseillevirus family
1.00
1.00
 Eukaryotes
0.57
 Eukaryotes
1.00
0.76
 Archaea
1.00
0.2
A
C
B
A
B
C
| Constraint 1 | E. huxleyi grouped with |
| --- | --- |
| Constraint 2 | E. huxleyi grouped with |
| Constraint 3 | E. huxleyi grouped with |
| Constraint 4 | Pandoraviruses grouped with |
| Constraint 5 | Pandoraviruses grouped with |
| Constraint 6 | Pandoraviruses grouped with |
A
B
C
Topology Testing results
| | elw | au | Likelihood |
| --- | --- | --- | --- |
| freetree | 0.79292 | 0.947844 | -91653.244 |
| constr1 | 0.06867 | 0.210901 | -91676.554 |
| constr2 | 0.017603 | 0.108029 | -91692.06 |
| constr3 | 0.004154 | 0 | -91717.672 |
| constr4 | 0.060846 | 0.19415 | -91676.759 |
| constr5 | 0.044414 | 0.084372 | -91706.579 |
| constr6 | 0.011398 | 0.111099 | -91692.75 |

## Slide 7
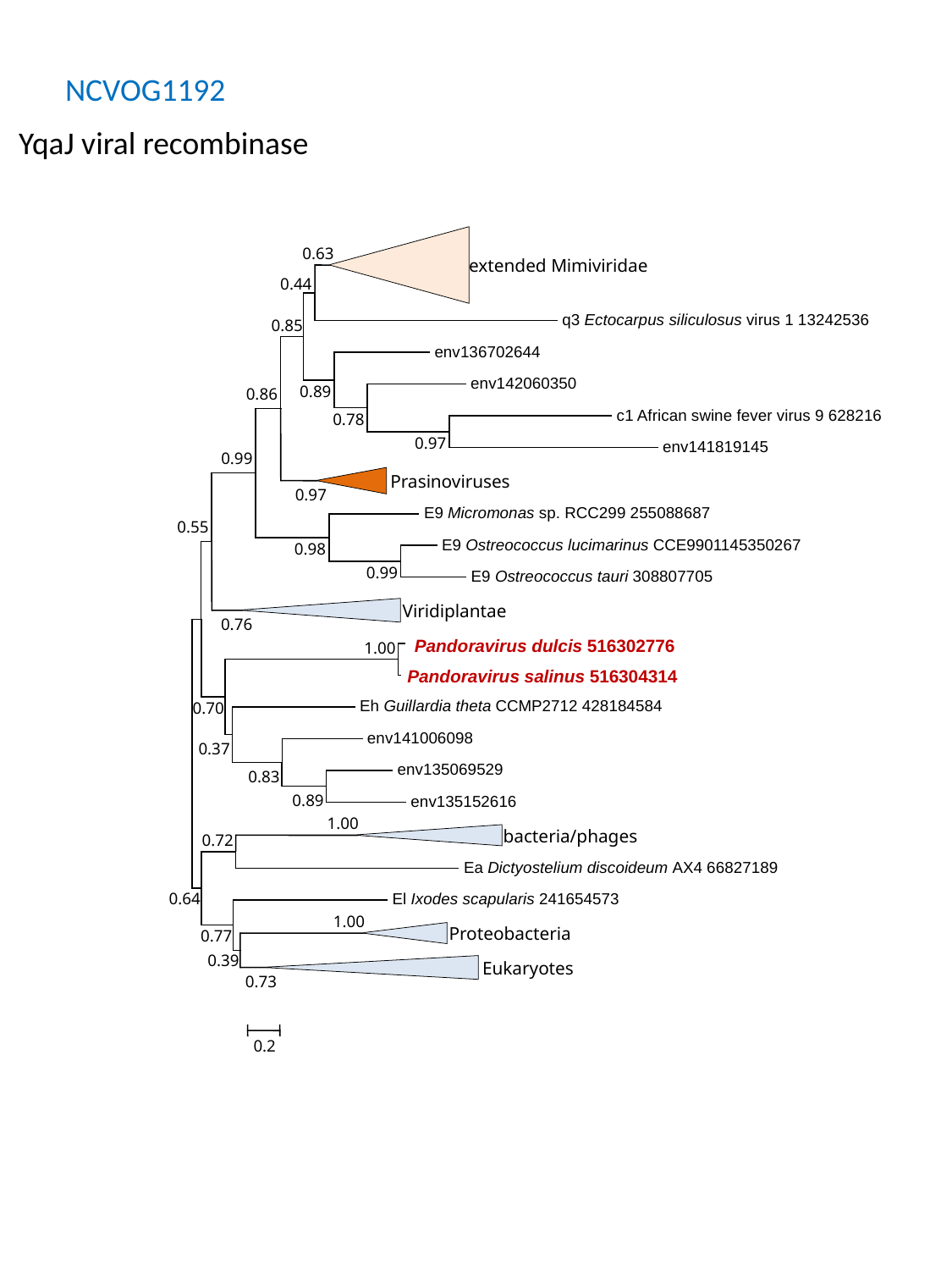

NCVOG1192
YqaJ viral recombinase
0.63
 extended Mimiviridae
0.44
 q3 Ectocarpus siliculosus virus 1 13242536
0.85
 env136702644
 env142060350
0.89
0.86
 c1 African swine fever virus 9 628216
0.78
0.97
 env141819145
0.99
 Prasinoviruses
0.97
 E9 Micromonas sp. RCC299 255088687
0.55
 E9 Ostreococcus lucimarinus CCE9901145350267
0.98
0.99
 E9 Ostreococcus tauri 308807705
 Viridiplantae
0.76
1.00
 Eh Guillardia theta CCMP2712 428184584
0.70
 env141006098
0.37
 env135069529
0.83
0.89
 env135152616
1.00
 bacteria/phages
0.72
 Ea Dictyostelium discoideum AX4 66827189
0.64
 El Ixodes scapularis 241654573
1.00
 Proteobacteria
0.77
0.39
 Eukaryotes
0.73
0.2
 Pandoravirus dulcis 516302776
 Pandoravirus salinus 516304314

## Slide 8
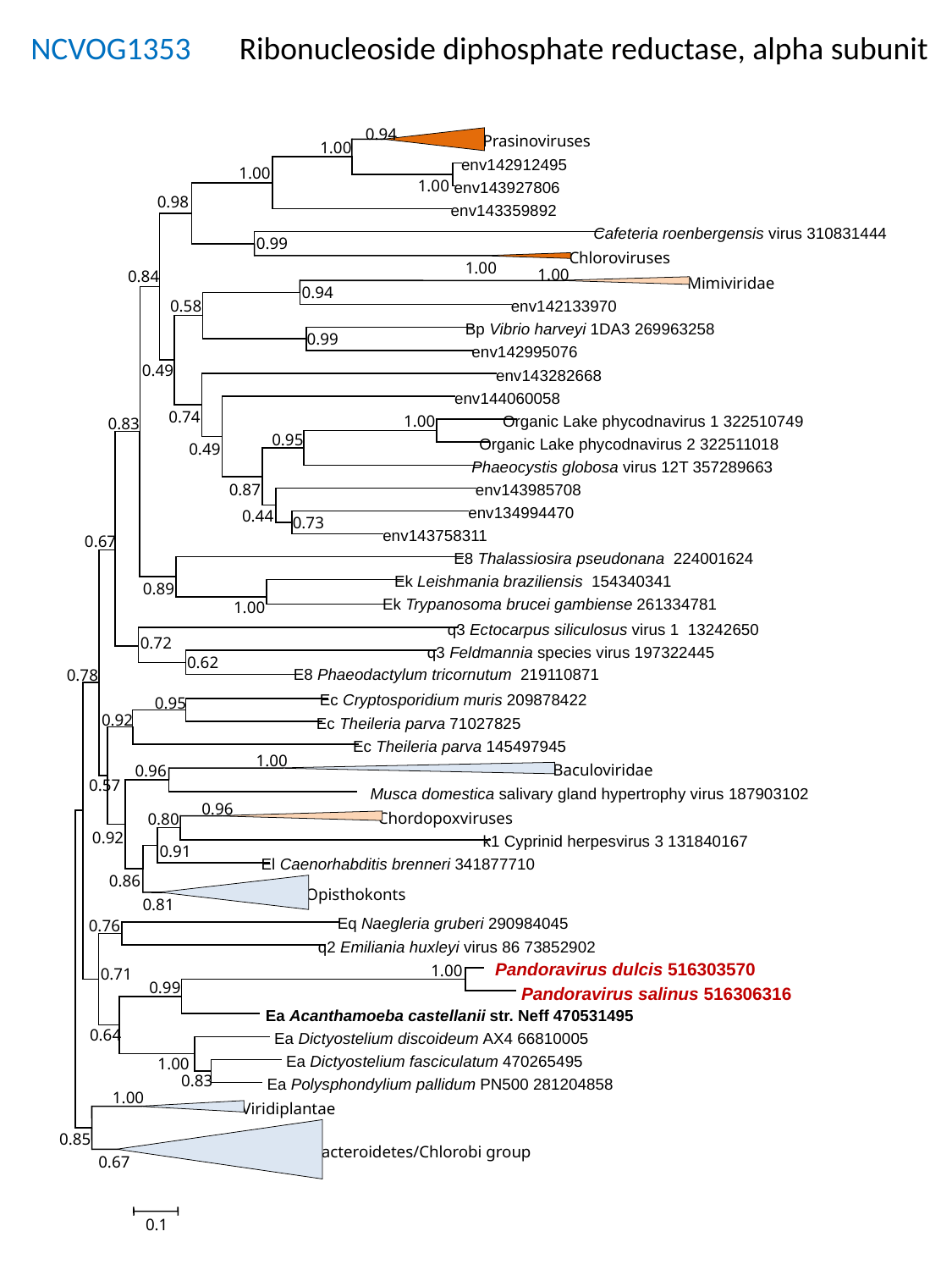

NCVOG1353
Ribonucleoside diphosphate reductase, alpha subunit
0.94
 Prasinoviruses
1.00
 env142912495
1.00
1.00
 env143927806
0.98
 env143359892
 Cafeteria roenbergensis virus 310831444
0.99
 Chloroviruses
1.00
1.00
0.84
 Mimiviridae
0.94
0.58
 env142133970
 Bp Vibrio harveyi 1DA3 269963258
0.99
 env142995076
0.49
 env143282668
 env144060058
0.74
Organic Lake phycodnavirus 1 322510749
1.00
0.83
0.95
 Organic Lake phycodnavirus 2 322511018
0.49
 Phaeocystis globosa virus 12T 357289663
0.87
 env143985708
 env134994470
0.44
0.73
 env143758311
0.67
 E8 Thalassiosira pseudonana 224001624
 Ek Leishmania braziliensis 154340341
0.89
 Ek Trypanosoma brucei gambiense 261334781
1.00
 q3 Ectocarpus siliculosus virus 1 13242650
0.72
 q3 Feldmannia species virus 197322445
0.62
 E8 Phaeodactylum tricornutum 219110871
0.78
 Ec Cryptosporidium muris 209878422
0.95
0.92
 Ec Theileria parva 71027825
 Ec Theileria parva 145497945
1.00
 Baculoviridae
0.96
0.57
 Musca domestica salivary gland hypertrophy virus 187903102
0.96
 Chordopoxviruses
0.80
0.92
 k1 Cyprinid herpesvirus 3 131840167
0.91
 El Caenorhabditis brenneri 341877710
0.86
 Opisthokonts
0.81
 Eq Naegleria gruberi 290984045
0.76
 q2 Emiliania huxleyi virus 86 73852902
 Pandoravirus dulcis 516303570
1.00
0.71
0.99
 Pandoravirus salinus 516306316
 Ea Acanthamoeba castellanii str. Neff 470531495
0.64
 Ea Dictyostelium discoideum AX4 66810005
 Ea Dictyostelium fasciculatum 470265495
1.00
0.83
 Ea Polysphondylium pallidum PN500 281204858
1.00
 Viridiplantae
0.85
 Bacteroidetes/Chlorobi group
0.67
0.1

## Slide 9
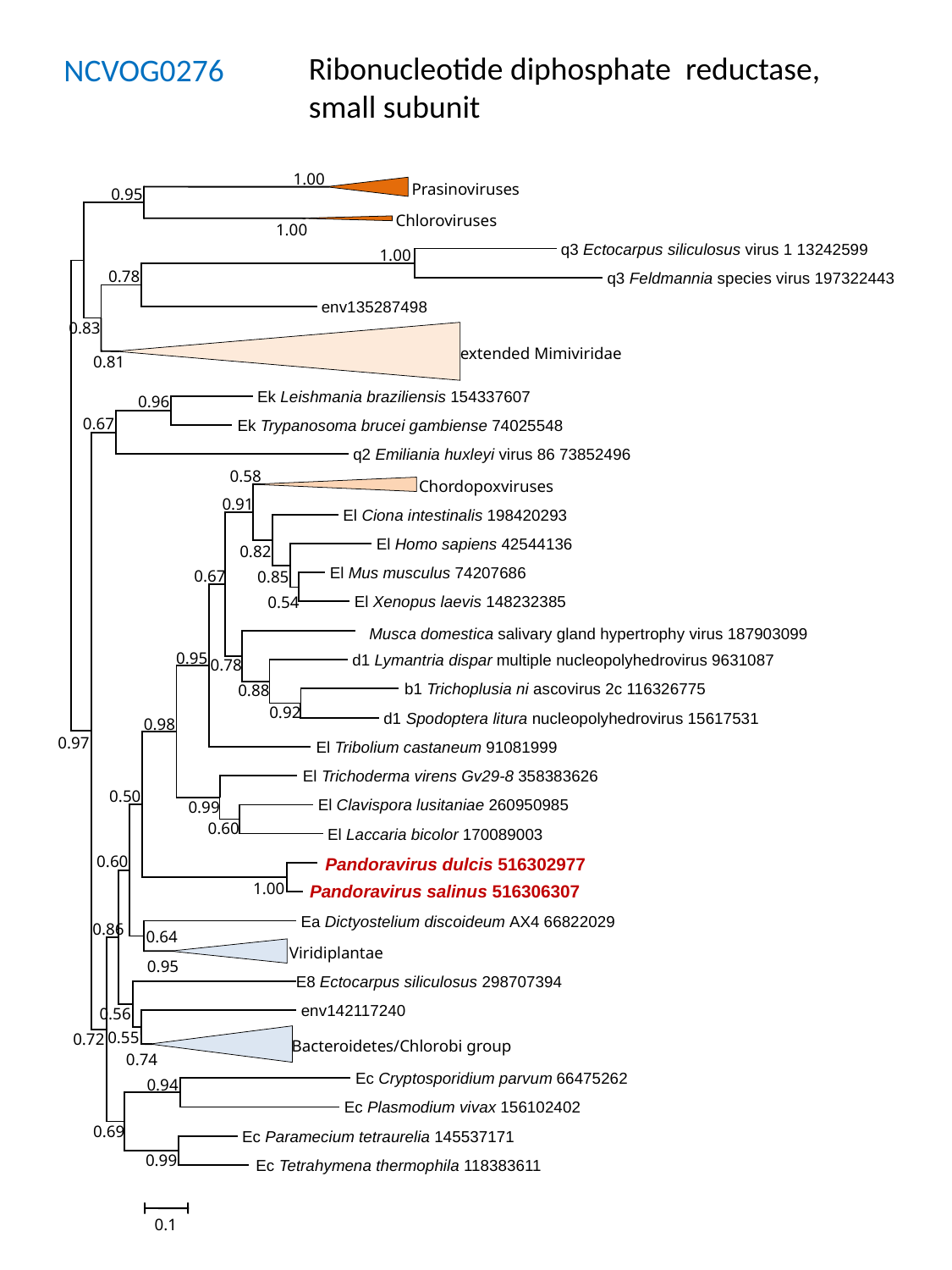

Ribonucleotide diphosphate reductase,
small subunit
NCVOG0276
1.00
 Prasinoviruses
0.95
 Chloroviruses
1.00
 q3 Ectocarpus siliculosus virus 1 13242599
1.00
0.78
 q3 Feldmannia species virus 197322443
 env135287498
0.83
 extended Mimiviridae
0.81
 Ek Leishmania braziliensis 154337607
0.96
0.67
 Ek Trypanosoma brucei gambiense 74025548
 q2 Emiliania huxleyi virus 86 73852496
0.58
 Chordopoxviruses
0.91
 El Ciona intestinalis 198420293
 El Homo sapiens 42544136
0.82
 El Mus musculus 74207686
0.67
0.85
 El Xenopus laevis 148232385
0.54
0.95
 d1 Lymantria dispar multiple nucleopolyhedrovirus 9631087
0.78
 b1 Trichoplusia ni ascovirus 2c 116326775
0.88
0.92
 d1 Spodoptera litura nucleopolyhedrovirus 15617531
0.98
0.97
 El Tribolium castaneum 91081999
 El Trichoderma virens Gv29-8 358383626
0.50
 El Clavispora lusitaniae 260950985
0.99
0.60
 El Laccaria bicolor 170089003
0.60
1.00
 Ea Dictyostelium discoideum AX4 66822029
0.86
0.64
 Viridiplantae
0.95
E8 Ectocarpus siliculosus 298707394
 env142117240
0.56
0.55
0.72
 Bacteroidetes/Chlorobi group
0.74
 Ec Cryptosporidium parvum 66475262
0.94
 Ec Plasmodium vivax 156102402
0.69
 Ec Paramecium tetraurelia 145537171
0.99
 Ec Tetrahymena thermophila 118383611
0.1
 Musca domestica salivary gland hypertrophy virus 187903099
 Pandoravirus dulcis 516302977
 Pandoravirus salinus 516306307

## Slide 10
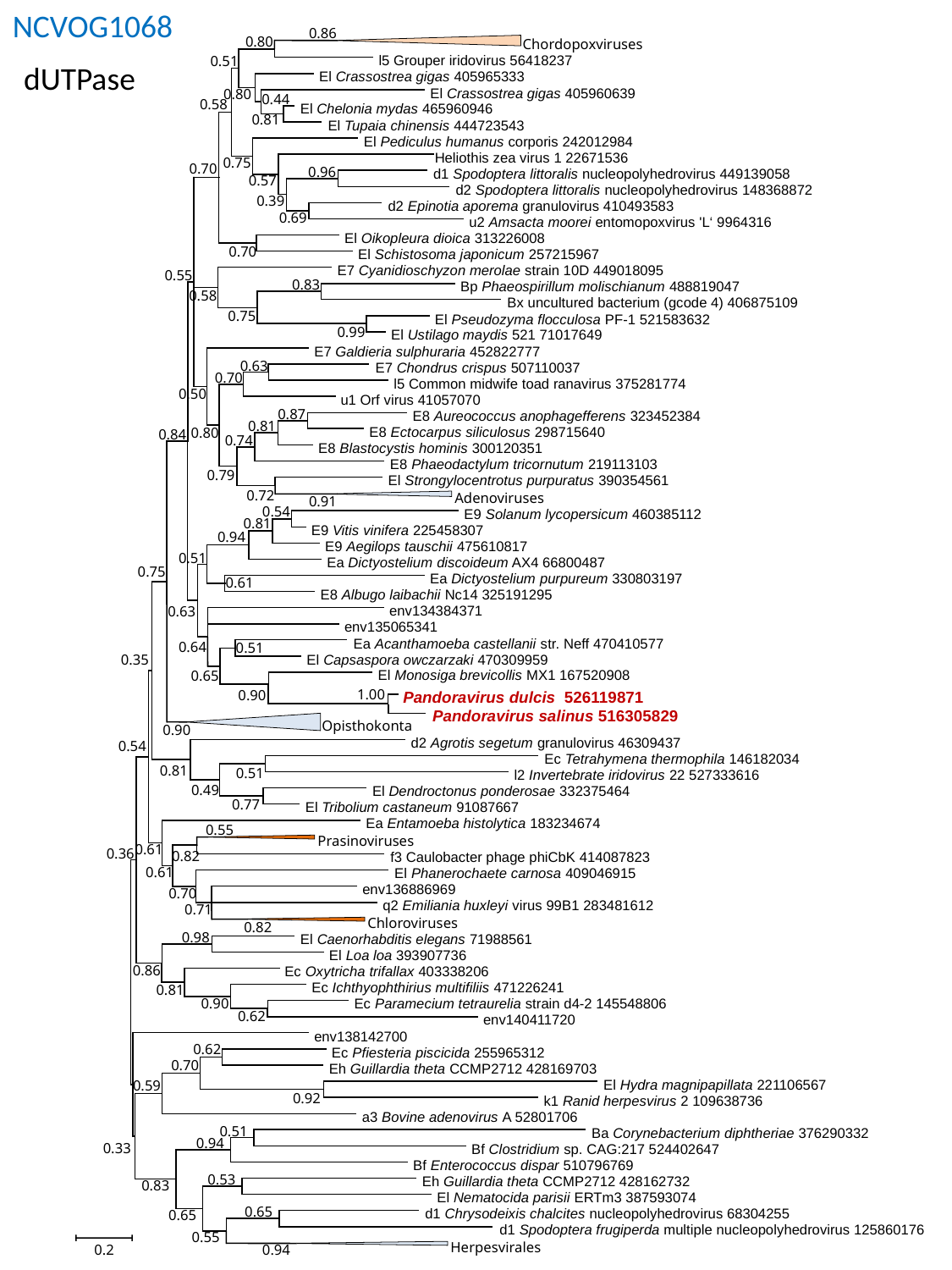

NCVOG1068
0.86
0.80
 Chordopoxviruses
 l5 Grouper iridovirus 56418237
0.51
 El Crassostrea gigas 405965333
 El Crassostrea gigas 405960639
0.80
0.44
0.58
 El Chelonia mydas 465960946
0.81
 El Tupaia chinensis 444723543
 El Pediculus humanus corporis 242012984
Heliothis zea virus 1 22671536
0.75
0.70
0.96
 d1 Spodoptera littoralis nucleopolyhedrovirus 449139058
0.57
 d2 Spodoptera littoralis nucleopolyhedrovirus 148368872
0.39
 d2 Epinotia aporema granulovirus 410493583
0.69
 u2 Amsacta moorei entomopoxvirus 'L‘ 9964316
 El Oikopleura dioica 313226008
0.70
 El Schistosoma japonicum 257215967
 E7 Cyanidioschyzon merolae strain 10D 449018095
0.55
0.83
 Bp Phaeospirillum molischianum 488819047
0.58
 Bx uncultured bacterium (gcode 4) 406875109
0.75
 El Pseudozyma flocculosa PF-1 521583632
0.99
 El Ustilago maydis 521 71017649
 E7 Galdieria sulphuraria 452822777
0.63
 E7 Chondrus crispus 507110037
0.70
 l5 Common midwife toad ranavirus 375281774
0.50
 u1 Orf virus 41057070
0.87
 E8 Aureococcus anophagefferens 323452384
0.81
 E8 Ectocarpus siliculosus 298715640
0.80
0.84
0.74
 E8 Blastocystis hominis 300120351
 E8 Phaeodactylum tricornutum 219113103
0.79
 El Strongylocentrotus purpuratus 390354561
0.72
 Adenoviruses
0.91
0.54
 E9 Solanum lycopersicum 460385112
0.81
 E9 Vitis vinifera 225458307
0.94
 E9 Aegilops tauschii 475610817
0.51
 Ea Dictyostelium discoideum AX4 66800487
0.75
 Ea Dictyostelium purpureum 330803197
0.61
 E8 Albugo laibachii Nc14 325191295
 env134384371
0.63
 env135065341
 Ea Acanthamoeba castellanii str. Neff 470410577
0.64
0.51
 El Capsaspora owczarzaki 470309959
0.35
 El Monosiga brevicollis MX1 167520908
0.65
1.00
0.90
 Pandoravirus dulcis 526119871
 Pandoravirus salinus 516305829
 Opisthokonta
0.90
 d2 Agrotis segetum granulovirus 46309437
0.54
 Ec Tetrahymena thermophila 146182034
0.81
0.51
 l2 Invertebrate iridovirus 22 527333616
0.49
 El Dendroctonus ponderosae 332375464
0.77
 El Tribolium castaneum 91087667
 Ea Entamoeba histolytica 183234674
0.55
 Prasinoviruses
0.61
0.36
0.82
 f3 Caulobacter phage phiCbK 414087823
0.61
 El Phanerochaete carnosa 409046915
 env136886969
0.70
 q2 Emiliania huxleyi virus 99B1 283481612
0.71
 Chloroviruses
0.82
0.98
 El Caenorhabditis elegans 71988561
 El Loa loa 393907736
0.86
 Ec Oxytricha trifallax 403338206
 Ec Ichthyophthirius multifiliis 471226241
0.81
 Ec Paramecium tetraurelia strain d4-2 145548806
0.90
0.62
 env140411720
 env138142700
0.62
 Ec Pfiesteria piscicida 255965312
0.70
 Eh Guillardia theta CCMP2712 428169703
 El Hydra magnipapillata 221106567
0.59
0.92
 k1 Ranid herpesvirus 2 109638736
 a3 Bovine adenovirus A 52801706
0.51
 Ba Corynebacterium diphtheriae 376290332
0.94
0.33
 Bf Clostridium sp. CAG:217 524402647
 Bf Enterococcus dispar 510796769
0.53
 Eh Guillardia theta CCMP2712 428162732
0.83
 El Nematocida parisii ERTm3 387593074
0.65
 d1 Chrysodeixis chalcites nucleopolyhedrovirus 68304255
0.65
 d1 Spodoptera frugiperda multiple nucleopolyhedrovirus 125860176
0.55
0.2
 Herpesvirales
0.94
dUTPase

## Slide 11
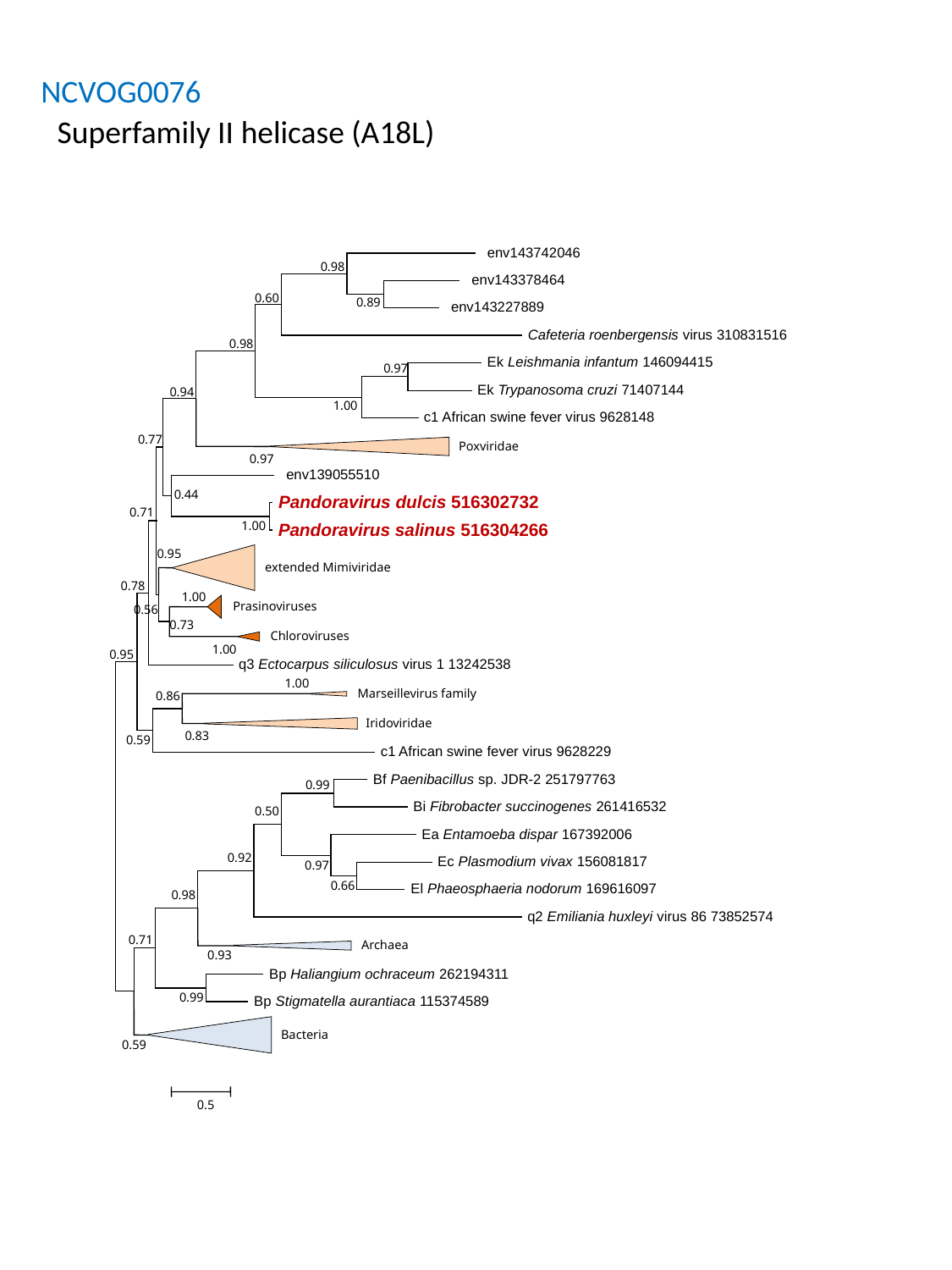

NCVOG0076
Superfamily II helicase (A18L)
 env143742046
0.98
 env143378464
0.60
0.89
 env143227889
 Cafeteria roenbergensis virus 310831516
0.98
 Ek Leishmania infantum 146094415
0.97
 Ek Trypanosoma cruzi 71407144
0.94
1.00
 c1 African swine fever virus 9628148
0.77
 Poxviridae
0.97
 env139055510
0.44
 Pandoravirus dulcis 516302732
0.71
1.00
 Pandoravirus salinus 516304266
0.95
 extended Mimiviridae
0.78
1.00
 Prasinoviruses
0.56
0.73
 Chloroviruses
1.00
0.95
 q3 Ectocarpus siliculosus virus 1 13242538
1.00
 Marseillevirus family
0.86
 Iridoviridae
0.83
0.59
 c1 African swine fever virus 9628229
 Bf Paenibacillus sp. JDR-2 251797763
0.99
 Bi Fibrobacter succinogenes 261416532
0.50
 Ea Entamoeba dispar 167392006
0.92
 Ec Plasmodium vivax 156081817
0.97
0.66
 El Phaeosphaeria nodorum 169616097
0.98
 q2 Emiliania huxleyi virus 86 73852574
0.71
 Archaea
0.93
 Bp Haliangium ochraceum 262194311
0.99
 Bp Stigmatella aurantiaca 115374589
 Bacteria
0.59
0.5

## Slide 12
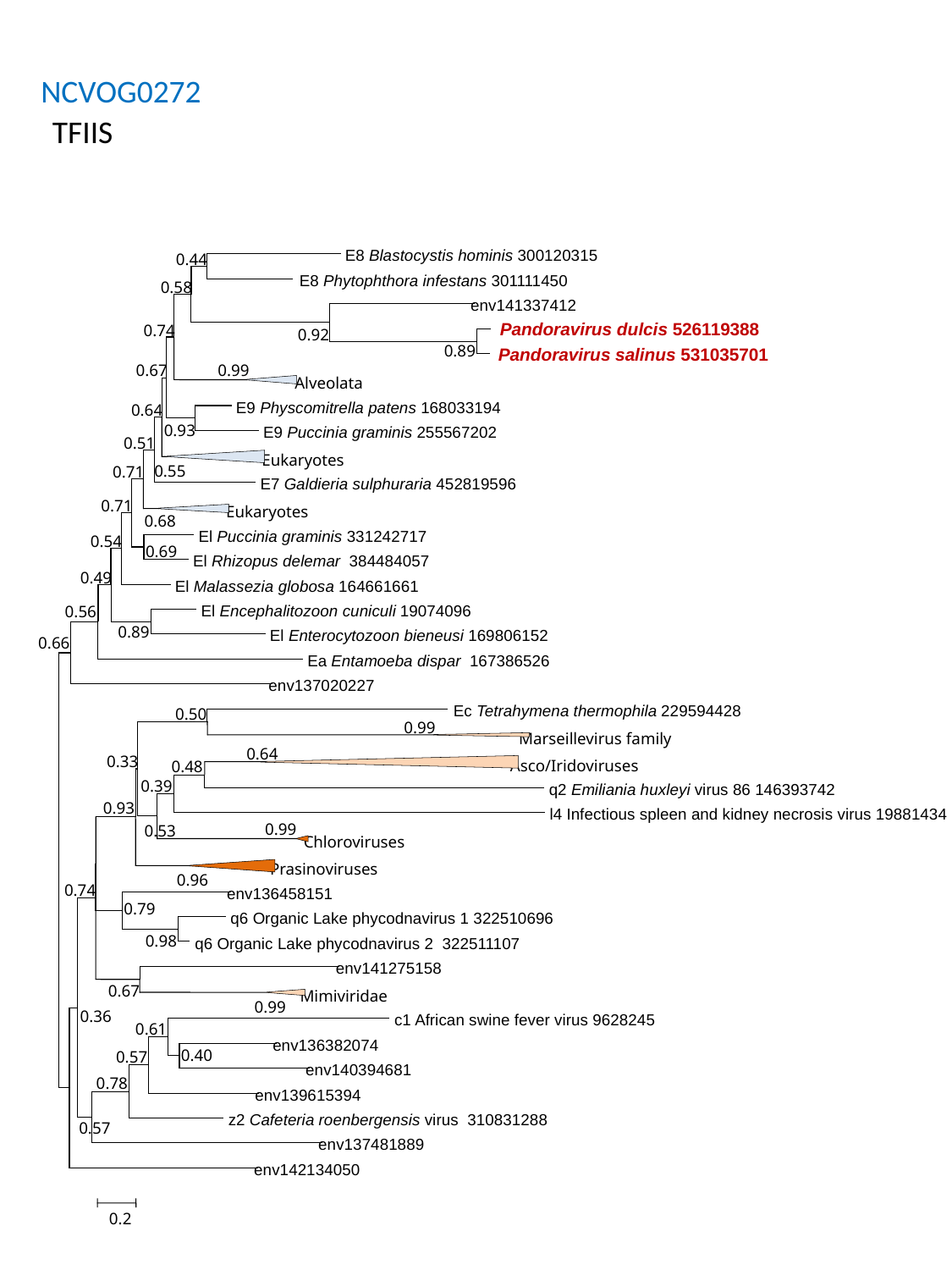

NCVOG0272
TFIIS
 E8 Blastocystis hominis 300120315
0.44
 E8 Phytophthora infestans 301111450
0.58
 env141337412
 Pandoravirus dulcis 526119388
0.74
0.92
0.89
 Pandoravirus salinus 531035701
0.99
0.67
 Alveolata
 E9 Physcomitrella patens 168033194
0.64
0.93
 E9 Puccinia graminis 255567202
0.51
 Eukaryotes
0.55
0.71
 E7 Galdieria sulphuraria 452819596
0.71
 Eukaryotes
0.68
 El Puccinia graminis 331242717
0.54
0.69
 El Rhizopus delemar 384484057
0.49
 El Malassezia globosa 164661661
 El Encephalitozoon cuniculi 19074096
0.56
0.89
 El Enterocytozoon bieneusi 169806152
0.66
 Ea Entamoeba dispar 167386526
 env137020227
 Ec Tetrahymena thermophila 229594428
0.50
0.99
 Marseillevirus family
0.64
0.33
 Asco/Iridoviruses
0.48
0.39
 q2 Emiliania huxleyi virus 86 146393742
0.93
 l4 Infectious spleen and kidney necrosis virus 19881434
0.99
0.53
 Chloroviruses
 Prasinoviruses
0.96
0.74
 env136458151
0.79
 q6 Organic Lake phycodnavirus 1 322510696
0.98
 q6 Organic Lake phycodnavirus 2 322511107
 env141275158
0.67
 Mimiviridae
0.99
0.36
 c1 African swine fever virus 9628245
0.61
 env136382074
0.40
0.57
 env140394681
0.78
 env139615394
 z2 Cafeteria roenbergensis virus 310831288
0.57
 env137481889
 env142134050
0.2

## Slide 13
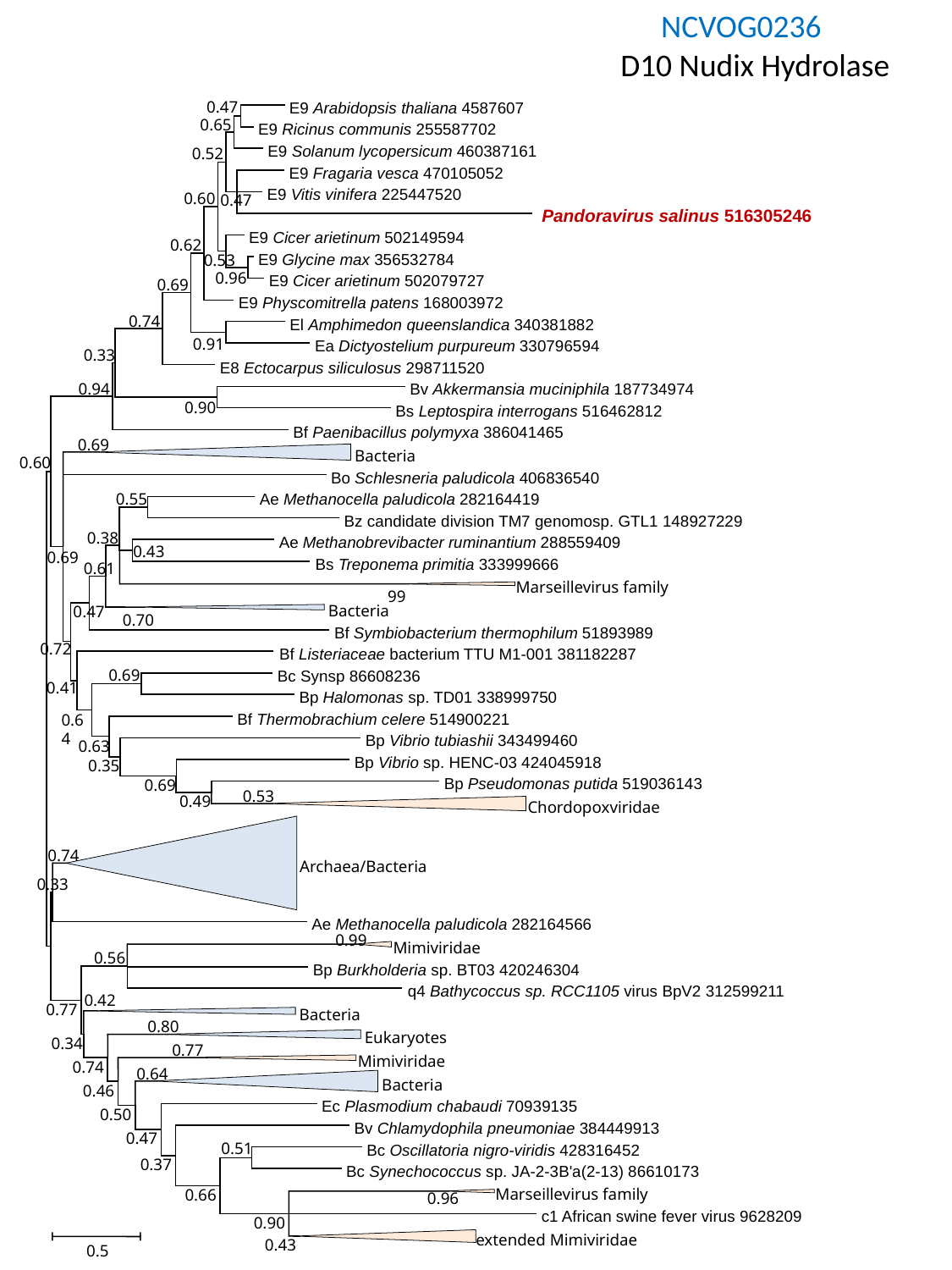

NCVOG0236
D10 Nudix Hydrolase
0.47
 E9 Arabidopsis thaliana 4587607
0.65
 E9 Ricinus communis 255587702
 E9 Solanum lycopersicum 460387161
0.52
 E9 Fragaria vesca 470105052
 E9 Vitis vinifera 225447520
0.60
0.47
 Pandoravirus salinus 516305246
 E9 Cicer arietinum 502149594
0.62
 E9 Glycine max 356532784
0.53
0.96
 E9 Cicer arietinum 502079727
0.69
 E9 Physcomitrella patens 168003972
0.74
 El Amphimedon queenslandica 340381882
0.91
 Ea Dictyostelium purpureum 330796594
0.33
 E8 Ectocarpus siliculosus 298711520
0.94
 Bv Akkermansia muciniphila 187734974
0.90
 Bs Leptospira interrogans 516462812
 Bf Paenibacillus polymyxa 386041465
0.69
 Bacteria
0.60
 Bo Schlesneria paludicola 406836540
0.55
 Ae Methanocella paludicola 282164419
 Bz candidate division TM7 genomosp. GTL1 148927229
0.38
 Ae Methanobrevibacter ruminantium 288559409
0.43
0.69
 Bs Treponema primitia 333999666
0.61
 Marseillevirus family
99
 Bacteria
0.47
0.70
 Bf Symbiobacterium thermophilum 51893989
0.72
 Bf Listeriaceae bacterium TTU M1-001 381182287
0.69
 Bc Synsp 86608236
0.41
 Bp Halomonas sp. TD01 338999750
 Bf Thermobrachium celere 514900221
0.64
 Bp Vibrio tubiashii 343499460
0.63
 Bp Vibrio sp. HENC-03 424045918
0.35
 Bp Pseudomonas putida 519036143
0.69
0.53
0.49
 Chordopoxviridae
0.74
 Archaea/Bacteria
0.33
 Ae Methanocella paludicola 282164566
0.99
 Mimiviridae
0.56
 Bp Burkholderia sp. BT03 420246304
 q4 Bathycoccus sp. RCC1105 virus BpV2 312599211
0.42
0.77
 Bacteria
0.80
 Eukaryotes
0.34
0.77
 Mimiviridae
0.74
0.64
 Bacteria
0.46
 Ec Plasmodium chabaudi 70939135
0.50
 Bv Chlamydophila pneumoniae 384449913
0.47
0.51
 Bc Oscillatoria nigro-viridis 428316452
0.37
 Bc Synechococcus sp. JA-2-3B'a(2-13) 86610173
 Marseillevirus family
0.66
0.96
 c1 African swine fever virus 9628209
0.90
 extended Mimiviridae
0.5
0.43

## Slide 14
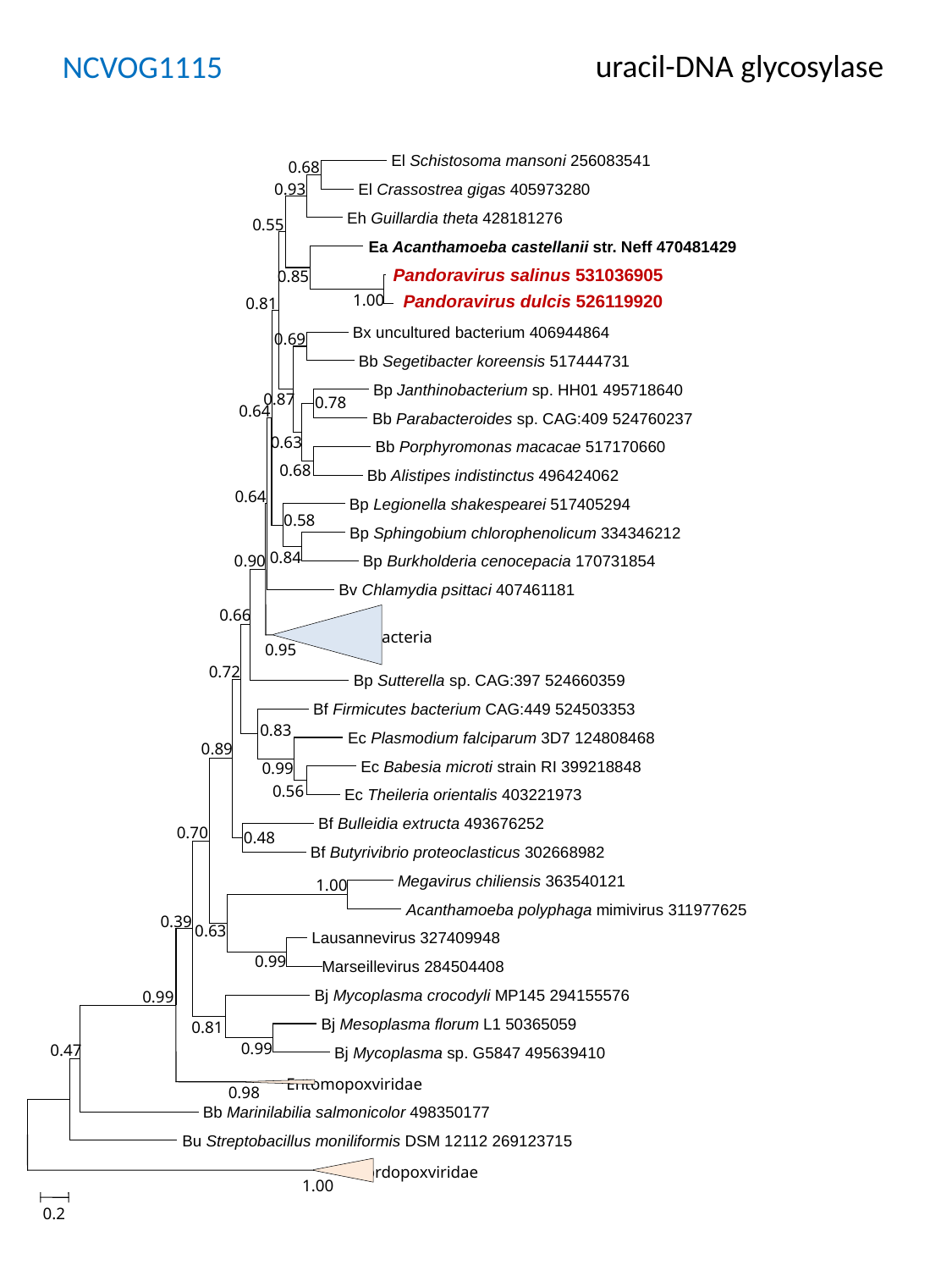

uracil-DNA glycosylase
NCVOG1115
 El Schistosoma mansoni 256083541
0.68
0.93
 El Crassostrea gigas 405973280
 Eh Guillardia theta 428181276
0.55
 Ea Acanthamoeba castellanii str. Neff 470481429
 Pandoravirus salinus 531036905
0.85
1.00
 Pandoravirus dulcis 526119920
0.81
 Bx uncultured bacterium 406944864
0.69
 Bb Segetibacter koreensis 517444731
 Bp Janthinobacterium sp. HH01 495718640
0.87
0.78
0.64
 Bb Parabacteroides sp. CAG:409 524760237
0.63
 Bb Porphyromonas macacae 517170660
0.68
 Bb Alistipes indistinctus 496424062
0.64
 Bp Legionella shakespearei 517405294
0.58
 Bp Sphingobium chlorophenolicum 334346212
0.84
0.90
 Bp Burkholderia cenocepacia 170731854
 Bv Chlamydia psittaci 407461181
0.66
 Bacteria
0.95
0.72
 Bp Sutterella sp. CAG:397 524660359
 Bf Firmicutes bacterium CAG:449 524503353
0.83
 Ec Plasmodium falciparum 3D7 124808468
0.89
 Ec Babesia microti strain RI 399218848
0.99
0.56
 Ec Theileria orientalis 403221973
 Bf Bulleidia extructa 493676252
0.70
0.48
 Bf Butyrivibrio proteoclasticus 302668982
 Megavirus chiliensis 363540121
1.00
 Acanthamoeba polyphaga mimivirus 311977625
0.39
0.63
 Lausannevirus 327409948
0.99
Marseillevirus 284504408
 Bj Mycoplasma crocodyli MP145 294155576
0.99
 Bj Mesoplasma florum L1 50365059
0.81
0.99
0.47
 Bj Mycoplasma sp. G5847 495639410
 Entomopoxviridae
0.98
 Bb Marinilabilia salmonicolor 498350177
 Bu Streptobacillus moniliformis DSM 12112 269123715
 Chordopoxviridae
1.00
0.2

## Slide 15
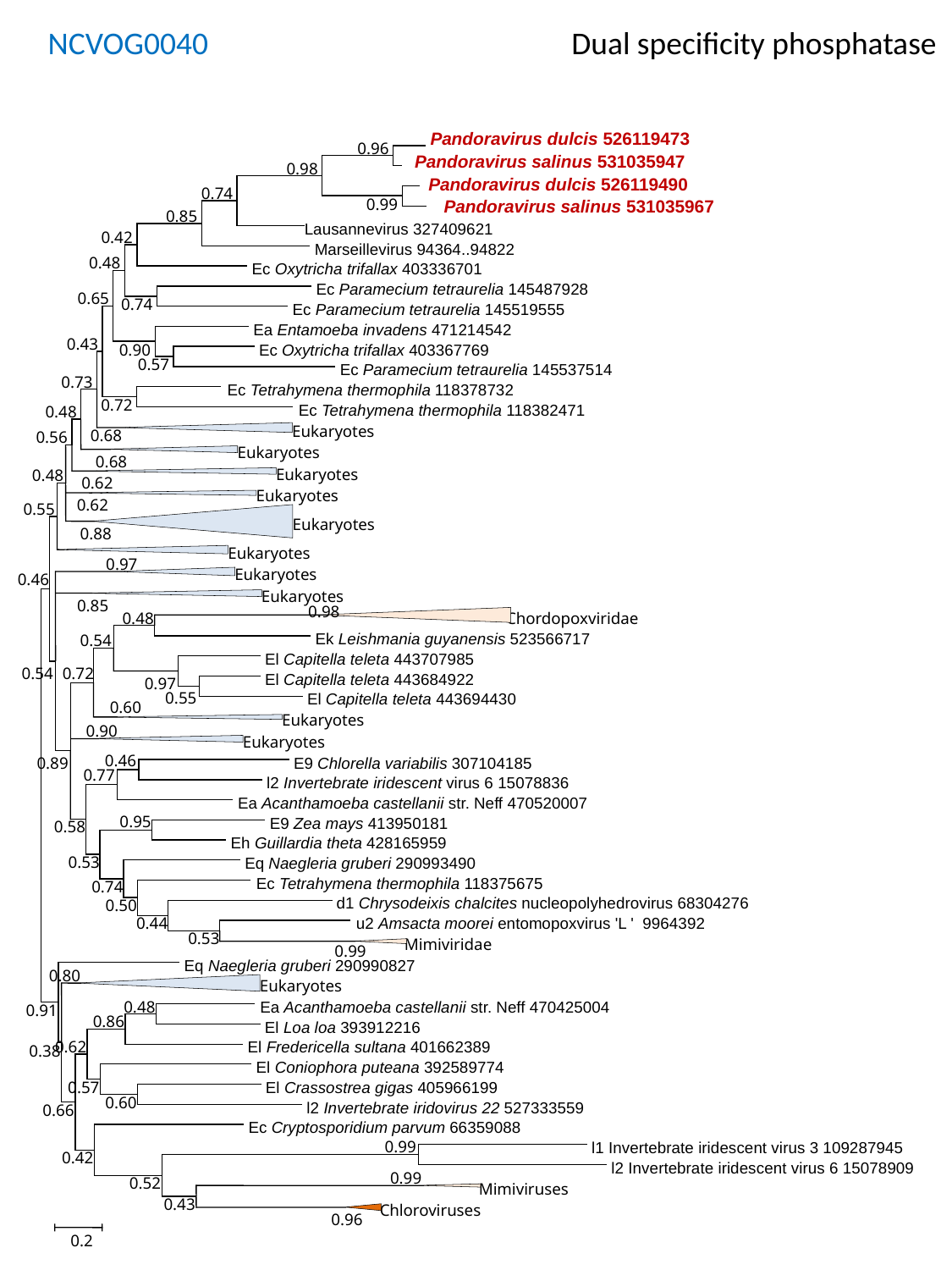

NCVOG0040
Dual specificity phosphatase
 Pandoravirus dulcis 526119473
0.96
 Pandoravirus salinus 531035947
0.98
 Pandoravirus dulcis 526119490
0.74
0.99
 Pandoravirus salinus 531035967
0.85
Lausannevirus 327409621
0.42
 Marseillevirus 94364..94822
0.48
 Ec Oxytricha trifallax 403336701
 Ec Paramecium tetraurelia 145487928
0.65
0.74
 Ec Paramecium tetraurelia 145519555
 Ea Entamoeba invadens 471214542
0.43
 Ec Oxytricha trifallax 403367769
0.90
0.57
 Ec Paramecium tetraurelia 145537514
0.73
 Ec Tetrahymena thermophila 118378732
0.72
 Ec Tetrahymena thermophila 118382471
0.48
 Eukaryotes
0.68
0.56
 Eukaryotes
0.68
 Eukaryotes
0.48
0.62
 Eukaryotes
0.62
0.55
 Eukaryotes
0.88
 Eukaryotes
0.97
 Eukaryotes
0.46
 Eukaryotes
0.85
0.98
0.48
 Chordopoxviridae
 Ek Leishmania guyanensis 523566717
0.54
 El Capitella teleta 443707985
0.72
0.54
 El Capitella teleta 443684922
0.97
0.55
 El Capitella teleta 443694430
0.60
 Eukaryotes
0.90
 Eukaryotes
0.46
 E9 Chlorella variabilis 307104185
0.89
0.77
 l2 Invertebrate iridescent virus 6 15078836
 Ea Acanthamoeba castellanii str. Neff 470520007
0.95
 E9 Zea mays 413950181
0.58
 Eh Guillardia theta 428165959
0.53
 Eq Naegleria gruberi 290993490
 Ec Tetrahymena thermophila 118375675
0.74
 d1 Chrysodeixis chalcites nucleopolyhedrovirus 68304276
0.50
0.44
 u2 Amsacta moorei entomopoxvirus 'L ' 9964392
0.53
 Mimiviridae
0.99
 Eq Naegleria gruberi 290990827
0.80
 Eukaryotes
0.48
 Ea Acanthamoeba castellanii str. Neff 470425004
0.91
0.86
 El Loa loa 393912216
0.62
 El Fredericella sultana 401662389
0.38
 El Coniophora puteana 392589774
 El Crassostrea gigas 405966199
0.57
0.60
 l2 Invertebrate iridovirus 22 527333559
0.66
 Ec Cryptosporidium parvum 66359088
0.99
 l1 Invertebrate iridescent virus 3 109287945
0.42
 l2 Invertebrate iridescent virus 6 15078909
0.99
0.52
 Mimiviruses
0.43
 Chloroviruses
0.96
0.2

## Slide 16
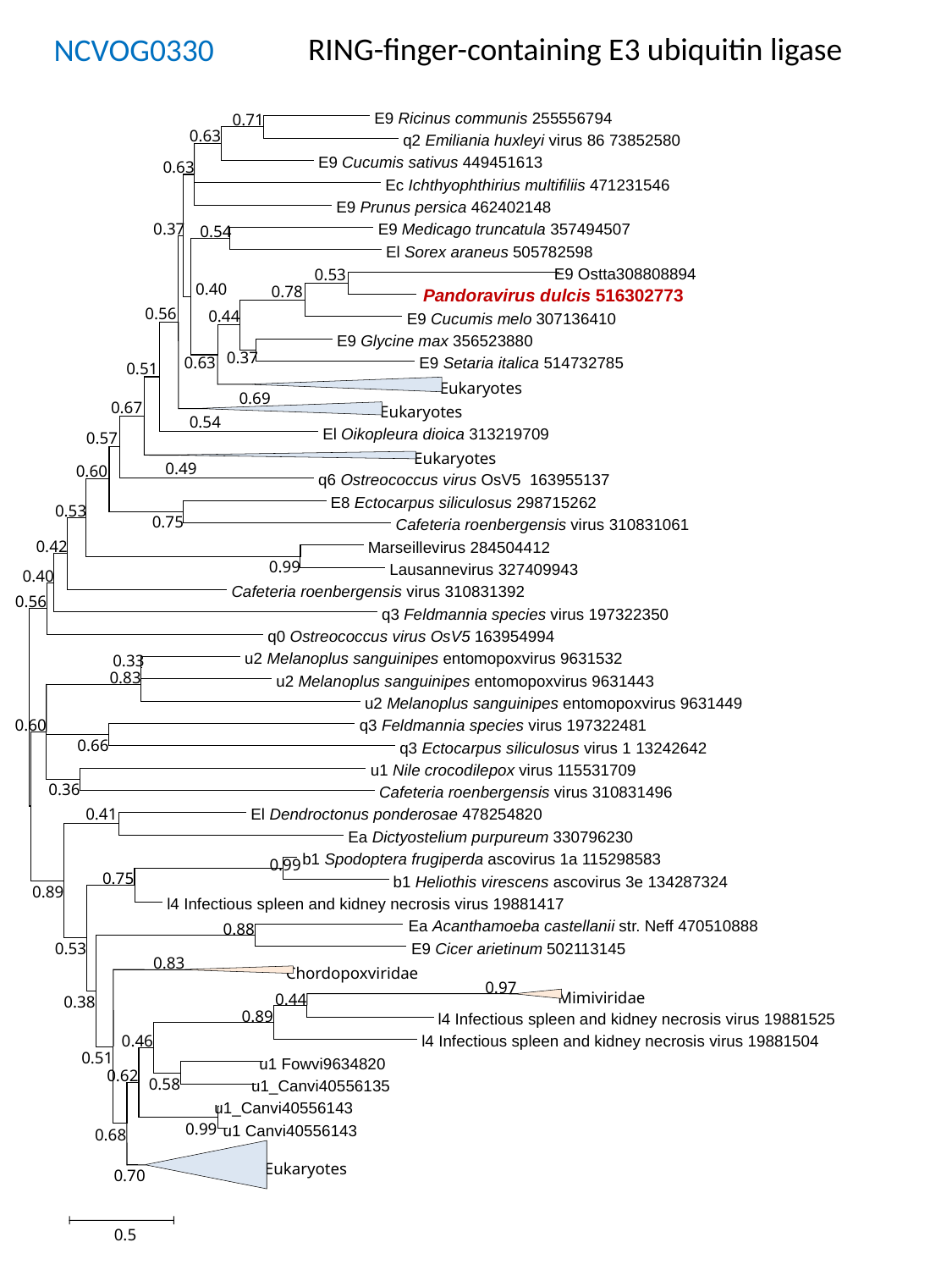

RING-finger-containing E3 ubiquitin ligase
NCVOG0330
 E9 Ricinus communis 255556794
0.71
0.63
 q2 Emiliania huxleyi virus 86 73852580
 E9 Cucumis sativus 449451613
0.63
 Ec Ichthyophthirius multifiliis 471231546
 E9 Prunus persica 462402148
0.37
 E9 Medicago truncatula 357494507
0.54
 El Sorex araneus 505782598
 E9 Ostta308808894
0.53
0.40
0.78
 Pandoravirus dulcis 516302773
0.56
0.44
 E9 Cucumis melo 307136410
 E9 Glycine max 356523880
0.37
0.63
 E9 Setaria italica 514732785
0.51
 Eukaryotes
0.69
0.67
 Eukaryotes
0.54
 El Oikopleura dioica 313219709
0.57
 Eukaryotes
0.49
0.60
 q6 Ostreococcus virus OsV5 163955137
 E8 Ectocarpus siliculosus 298715262
0.53
0.75
 Cafeteria roenbergensis virus 310831061
0.42
 Marseillevirus 284504412
0.99
 Lausannevirus 327409943
0.40
 Cafeteria roenbergensis virus 310831392
0.56
 q3 Feldmannia species virus 197322350
 q0 Ostreococcus virus OsV5 163954994
 u2 Melanoplus sanguinipes entomopoxvirus 9631532
0.33
0.83
 u2 Melanoplus sanguinipes entomopoxvirus 9631443
 u2 Melanoplus sanguinipes entomopoxvirus 9631449
0.60
 q3 Feldmannia species virus 197322481
0.66
 q3 Ectocarpus siliculosus virus 1 13242642
 u1 Nile crocodilepox virus 115531709
0.36
 Cafeteria roenbergensis virus 310831496
0.41
 El Dendroctonus ponderosae 478254820
 Ea Dictyostelium purpureum 330796230
 b1 Spodoptera frugiperda ascovirus 1a 115298583
0.99
0.75
 b1 Heliothis virescens ascovirus 3e 134287324
0.89
 l4 Infectious spleen and kidney necrosis virus 19881417
 Ea Acanthamoeba castellanii str. Neff 470510888
0.88
 E9 Cicer arietinum 502113145
0.53
0.83
 Chordopoxviridae
0.97
 Mimiviridae
0.44
0.38
0.89
 l4 Infectious spleen and kidney necrosis virus 19881525
0.46
 l4 Infectious spleen and kidney necrosis virus 19881504
0.51
 u1 Fowvi9634820
0.62
0.58
 u1_Canvi40556135
 u1_Canvi40556143
0.99
 u1 Canvi40556143
0.68
 Eukaryotes
0.70
0.5

## Slide 17
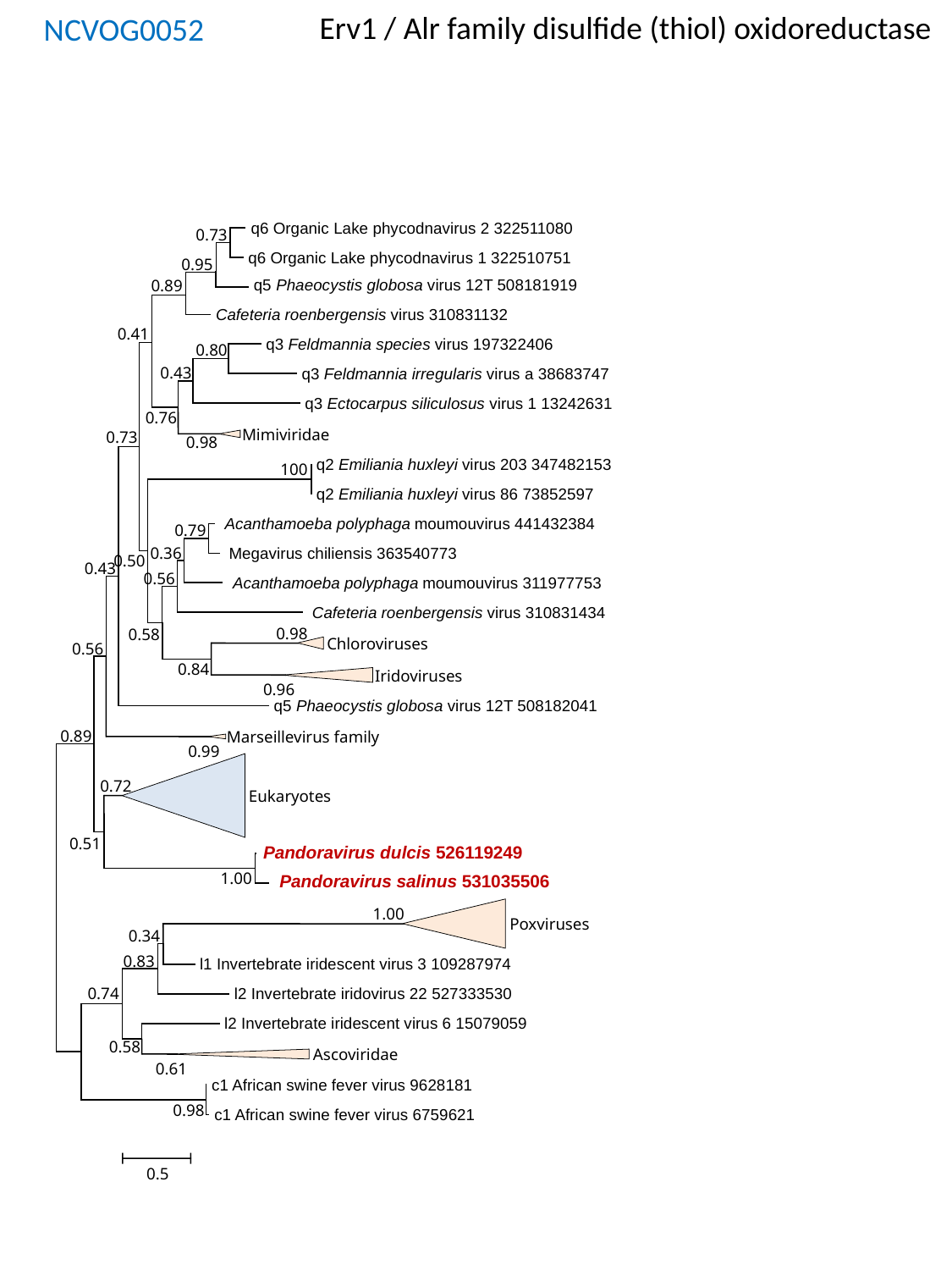

Erv1 / Alr family disulfide (thiol) oxidoreductase
NCVOG0052
 q6 Organic Lake phycodnavirus 2 322511080
0.73
 q6 Organic Lake phycodnavirus 1 322510751
0.95
 q5 Phaeocystis globosa virus 12T 508181919
0.89
 Cafeteria roenbergensis virus 310831132
0.41
 q3 Feldmannia species virus 197322406
0.80
0.43
 q3 Feldmannia irregularis virus a 38683747
 q3 Ectocarpus siliculosus virus 1 13242631
0.76
 Mimiviridae
0.73
0.98
 q2 Emiliania huxleyi virus 203 347482153
100
 q2 Emiliania huxleyi virus 86 73852597
 Acanthamoeba polyphaga moumouvirus 441432384
0.79
0.36
 Megavirus chiliensis 363540773
0.50
0.43
0.56
 Acanthamoeba polyphaga moumouvirus 311977753
 Cafeteria roenbergensis virus 310831434
0.98
0.58
 Chloroviruses
0.56
0.84
 Iridoviruses
0.96
 q5 Phaeocystis globosa virus 12T 508182041
0.89
 Marseillevirus family
0.99
0.72
 Eukaryotes
0.51
 Pandoravirus dulcis 526119249
1.00
 Pandoravirus salinus 531035506
1.00
 Poxviruses
0.34
0.83
 l1 Invertebrate iridescent virus 3 109287974
0.74
 l2 Invertebrate iridovirus 22 527333530
 l2 Invertebrate iridescent virus 6 15079059
0.58
 Ascoviridae
0.61
 c1 African swine fever virus 9628181
0.98
 c1 African swine fever virus 6759621
0.5
